# Breaking barriers: exploring mechanisms behind opening the blood–brain barrier

**DOI:** 10.1186/s12987-023-00489-2

**Published:** 2023-11-28

**Authors:** Melanie E. M. Stamp, Michael Halwes, David Nisbet, David J. Collins

**Affiliations:** 1 Department of Biomedical Engineering, The University of Melbourne, Melbourne, Australia; 2 Graeme Clark Institute for Biomedical Engineering, The University of Melbourne, Melbourne, Australia

**Keywords:** Blood–Brain barrier, Tight junctions, BBB stimulation, Focused ultrasound, Neurological therapeutics

## Abstract

The blood–brain barrier (BBB) is a selectively permeable membrane that separates the bloodstream from the brain. While useful for protecting neural tissue from harmful substances, brain-related diseases are difficult to treat due to this barrier, as it also limits the efficacy of drug delivery. To address this, promising new approaches for enhancing drug delivery are based on disrupting the BBB using physical means, including optical/photothermal therapy, electrical stimulation, and acoustic/mechanical stimulation. These physical mechanisms can temporarily and locally open the BBB, allowing drugs and other substances to enter. Focused ultrasound is particularly promising, with the ability to focus energies to targeted, deep-brain regions. In this review, we examine recent advances in physical approaches for temporary BBB disruption, describing their underlying mechanisms as well as evaluating the utility of these physical approaches with regard to their potential risks and limitations. While these methods have demonstrated efficacy in disrupting the BBB, their safety, comparative efficacy, and practicality for clinical use remain an ongoing topic of research.

## Background

A brain’s structure and environment, including the cellular microenvironment, must be tightly controlled for it to properly function [1]. The architecture and organisation of brain vasculature are vital to maintaining this microenvironment [2]. Numerous major arteries branch out into increasingly smaller blood vessels, ending in a dense network of capillaries. This capillary network accounts for 85% of the surface area between the bloodstream and the brain parenchyma, the latter comprising neurons and glial cells [2, 3]. Blood flow from these cells is separated by the blood–brain barrier (BBB), an efficient and highly selective permeable barrier. Many neurological disorders are caused by dysfunction and breakdown of the BBB [4–6]. At other times, the selective barrier hampers the delivery of therapeutic drugs into brain parenchyma targets. Therefore, medical researchers are increasingly interested in technologies that help them not only better understand the BBB but also circumvent the BBB to deliver drugs to targeted brain regions [3, 7–9]. On the topic of circumventing the BBB, recent research has focused on the dynamics regulating the BBB and the effects of its disruption [10, 11], where understanding these processes can help identify therapeutic targets. An important finding of this work is that molecular waste compounds across the BBB in many neurodegenerative diseases [12–14] and directly contribute to disease progression [15]. Conversely, several therapeutic strategies aim to intentionally and temporarily increase BBB permeability to improve drug delivery, since many pharmaceuticals cannot cross an intact BBB [8, 16, 17]. Such strategies often employ chemical methods, such as modifying therapeutic agents’ chemical structure to exploit endogenous transport mechanisms or encapsulating them in nanoparticle vehicles to cross the BBB. Despite extensive research on chemical-based approaches, their clinical applications remain limited, with high failure rates in clinical trials [3, 14]. A promising alternative strategy, however, involves physically altering the BBB’s structure and function to temporarily allow larger molecules to pass. Paracellular transport can be enhanced by modulating cell function to disrupting tight junctions [18–20], multiprotein complexes that bind adjoining endothelial cells in vasculature lumens.

Here, we examine the physical mechanisms and the efficacy of three common strategies for modulating BBB transport: optical/photothermal therapy, electrical stimulation, and acoustic/mechanical stimulation. At a basic level, BBB modulation depends on modifying the physical gaps proteins that bind adjacent cells, hence, tight junction proteins and paracellular transport are of particular interest. Next, we analyse the three stimulation approaches in turn, highlighting work from in vivo and in vitro studies that shed light on their modulation mechanisms. We conclude by summarising key differences between these approaches, considerations for clinical application, and possible future directions.

### Anatomy of the BBB

#### Cellular components of the BBB

**Fig. 1:**
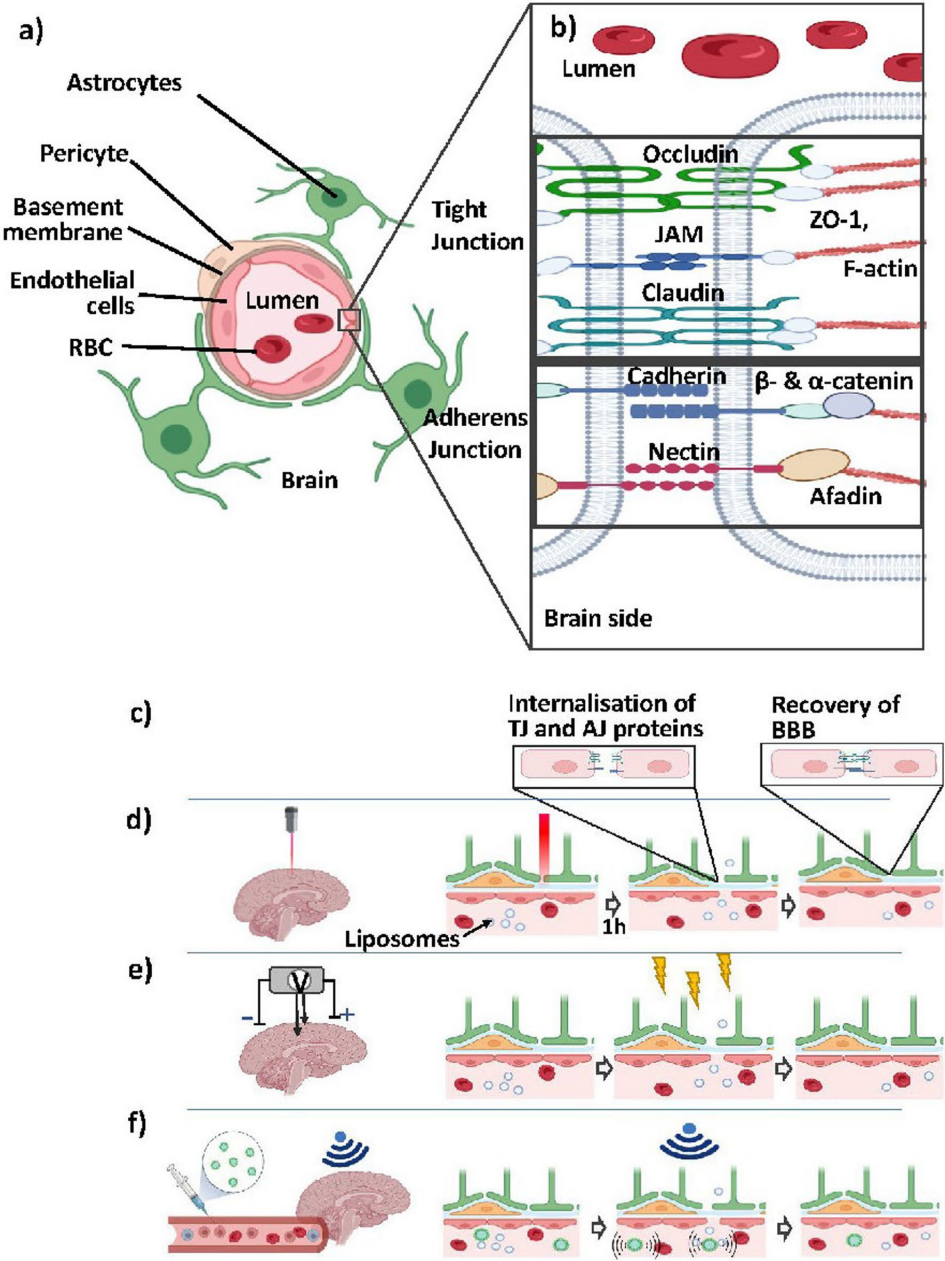
Schematic of a BBB. **a** Cell components. A basement membrane surrounds the endothelial cells that form the lumen. Surrounding these are pericytes and astrocytes, which together cover roughly 30 and 99% of the blood vessel, respectively. **b** Tight and adherens junctions. Apical and lumen TJs are composed of three transmembrane-spanning proteins: Occludin, Claudin, and JAM, which recruit ZO-1 (ZO-2, ZO-3), an actin-binding protein. Adjoining are AJs, which are composed of nectin- and cadherin-based adhesions. In the extracellular domain, nectins of neighbouring cells dimerise while the cytoplasmic tail recruits Afadin. The cadherin cytoplasmic tail recruits β-catenin, which binds to α-catinen that connects to the F-actin. **c** Schematics of BBB opening process during stimulation. TJ and AJ proteins internalise and retract to open the BBB for liposomes to trespass before recovery. **d** In photodynamic therapy, in which the brain is exposed to light for a certain time, post-exposure BBB opening, and subsequent recovery is observed. **e** Process in electroporation and non-invasive transcranial electrodes. During exposure to an electric field, TJ & AJ proteins functions reduce, allowing particles to pass paracellularly. Removal of stimulation leads to an almost instant BBB closure. **f** schematic of FUS with microbubbles injected intravenously prior to stimulation. During FUS stimulation, microbubbles within the capillaries are excited via external US, with vibrations opening the BBB, where removal of US leads to immediate closure of the BBB

To understand transport mechanisms across the BBB, we first give an overview regarding its structural and functional components, detailing the cell types involved and their roles in BBB regulation (Fig. 1a). Endothelial cells (ECs) form the walls of the blood vessels by curving to form a lumen, resulting in walls as thin as 0.1 μm [21]. ECs are connected by adherens junctions and tight junctions, and together, they secrete the 30–40 nm thick vascular basement membrane, composed of collagen IV, heparan sulphate, laminin, fibronectin, and other extracellular matrix proteins [22]. To facilitate blood-parenchyma exchange, ECs express numerous receptors and transporters. Pro-inflammatory signals cause ECs to release cytokines that attract circulating leukocytes [23]. In the central nervous system, ECs are enriched with tight junctions, giving the brain microvasculature its non-fenestrated appearance. While other cells play a role in the BBB, the ECs form the most significant component of the BBB environment and function.

Partially enveloping ECs, pericytes sit embedded within the vascular basement membrane, providing intercellular signals regulating, EC development and behaviour. They extend long membrane processes across the abluminal side of capillaries [24] and cover about 22–37% of the EC surface [25] in an endothelial cell-to-pericyte ratio of approximately 3:1. A lack of pericytes correlates with increased BBB permeability via upregulation of endothelial transcytosis, suggesting an important role of pericytes in maintaining the integrity of the BBB [26, 27]. Additionally, pericytes regulate angiogenesis, deposit extracellular matrix components, and regulate the infiltration of immune cells [28].

Astrocytes further encapsulate blood vessels in the brain, extending their end-feet over 99% of the endothelial surface [25]. Astrocytes secrete their own parenchymal basement membrane in the perivascular space, also called the glia limitans [15]. Astrocytes promote BBB formation and integrity via the Hedgehog signalling pathway [29]. Other astrocyte functions include monitoring electrochemical activity, regulating innate immunity and balancing parenchymal water and metabolites [30, 31]. This cellular arrangement is coated by an extracellular matrix, which accounts for 20% of the BBB volume [25].

#### Endothelial cell junctions and the BBB

Through junctional protein complexes, endothelial cells reduce diffusion and maintain mechanical stability. In this section, we examine the configuration and function of junctional complex components. Tight junctions (TJ) are found near the apical (lumen side) part of the cell, conjoined to the adherens junctions (AJ) [32]. Figure 1b depicts a schematic of the junctional complex and proteins involved, which are also listed in Table 1. By interacting with the cytoskeleton, adherens junctions link ECs to form continuous sheets and drive tissue morphogenesis [33]. Adherens junctions are formed by the homophilic interactions between transmembrane cadherin proteins and intracellular catenin proteins to anchor them to actin filaments and tubulin microtubules [34, 35]. Cadherin-10 is predominately expressed in brain microvessels with BBB phenotypes, while VE-cadherin is more prevalent in larger pial vessels and in leakier barriers [36]. β-catenin binds and stabilises the cytoplasmic cadherin tail, associating with α-catenin to anchor to actin filaments. Attachment to microtubules is facilitated by p120 catenin. Cadherins and catenins mediate processes such as pericyte interactions and barrier integrity [37].

**Table 1:** Tight and adherens junction proteins involved in BBB opening

Junction protein	Position	Role in BBB opening
Claudin	TJ, Transmembrane	Structural integrity/permeability of the TJ, TJ assembly
Occludin	TJ, Transmembrane	Regulates permeability
Zonula occludens (ZO)	TJ, Intracellular	Anchors occludin and claudin to actin cytoskeleton
JAM	TJ, Transmembrane	Guides tight junction assembly
P-glycoprotein	TJ, Transmembrane	Efflux transporter
VE-cadherin	AJ, Transmembrane	Forms adherens junction, cell adhesion and metabolism
Nectin	AJ, Transmembrane	Forms adherens junctions, Leukocyte trafficking
α/β-catenin	AJ, Intracellular	Anchors VE-cadherin to actin cytoskeleton
afadin	AJ, Intracellular	Anchors Nectin to actin cytoskeleton

Tight junctions (TJ) play a significant role in regulating the endothelial microenvironment by regulating the passage of solutes between cells and by restricting the diffusion of membrane proteins between the apical and basal cell surfaces [37]. The BBB has a characteristically high trans-endothelial electrical resistance (TEER) due to limited paracellular transport caused by tight junctions. Examining these junctions in more detail, TJs are formed by three types of transmembrane proteins, namely occludin claudins, and junctional adhesion molecules and anchored to the actin cytoskeleton via cytoplasmic scaffolding proteins (ZO-1, ZO-2, and ZO-3) and heterotrimeric G-proteins [38]. It is believed that occludin plays the most important role in regulating permeability of TJs, though it is not essential for TJ formation [38, 39]. Claudins, on the other hand, are the primary constituents of the TJ strands linking the membranes of adjacent cells. There are 27 known claudins, and while claudin expression varies between species and cell type, claudin-1, -5, -11, -12, -25, and -27 have been found at comparably high levels in the human brain microvasculature [40]. This, alongside earlier studies into claudin-5 deficient mice showing intact TJs, supports the idea that these proteins can compensate for one another to a certain extent. [41, 42]. JAMs are believed to localise ZO-1 and occludin to TJ complexes [43]. Further, although TJs and AJs are distinct structures, there is significant crosstalk between the processes governing their expression and maturity [37]. More detailed information regarding the interplay between these junctions can be found in Campbell et al. [32].

#### Physiological blood brain barrier transport

TJs in the BBB limit passive diffusion through the paracellular space, meaning that only molecules with specific characteristics can pass through. Molecules are limited to a maximum molecular weight of 400–450 Da [44, 45] and must be sufficiently lipophilic [46]. Transcellular routes also allow molecules to enter and leave the parenchyma via a variety of mechanisms [30, 47]. In carrier-mediated transport, larger molecules are transported across the membrane by proteins (e.g., GLUT-1). Active efflux, for example, is the pumping out of brain materials by ATP-binding cassette transporters, which consume energy. Relevant examples include P-glycoprotein and Breast Cancer Resistance Protein [48]. The ECs can also transport larger molecules through endocytosis, encapsulating them in caveolin-lined vesicles. This process of transcytosis can be either specific (i.e., receptor-mediated) or non-specific (i.e., adsorption-mediated). Several proteins are involved in receptor-mediated transcytosis relevant to neurodegenerative diseases, including LDL-receptor-related protein-1 and the receptor for advanced glycation end-products (RAGE) [46, 49]. Endothelial membranes also contain numerous ion channels, co-transporters, and pumps that maintain electrochemical homeostasis [50]. For a more in-depth understanding of cellular junctions in the BBB and the various transport mechanisms, the interested reader is directed to the following reviews on the topic [30, 33, 38, 50].

## Opening the blood brain barrier—physical stimulation methods

In recent years, researchers have demonstrated methods of circumventing the BBB, often with the goal of increasing the accumulation of therapeutic agents in the parenchyma. Generally, these methods can be categorised as transcellular and paracellular. In transcellular methods, molecules are either made more lipophilic for passive diffusion, or they are transported through endothelial cells by carrier-mediated transport or receptor-mediated transcytosis [51]. Nevertheless, transcellular methods require use of a limited number of compatible pharmaceuticals [48]. Alternatively, paracellular methods weaken tight and adherens junctions, allowing molecules to migrate between cells.

Paracellular methods of bypassing the BBB involve both chemical and physical mechanisms. Chemical mechanisms [1, 52, 53] often utilise vasoactive compounds such as histamine, bradykinin, alkylglycerols, tumour necrosis factor, or interferon-γ to activate signalling pathways within endothelial cells, ultimately resulting in increased BBB permeability [54]. Alternatives include hyperosmolar agents such as mannitol to reduce endothelial intracellular volume, opening the BBB. However, chemical approaches often produce off-target effects and tissue damage, limiting their clinical utility [48, 54, 55]. The controlled opening of the BBB has been demonstrated through the use of artificial molecules, such as antibodies or peptides, which interact with the claudin family. For example, claudin-5 antibodies enhanced drug penetration into the BBB [56–58]. However, as they are distributed throughout the entire brain, these binding-modulator approaches still lack spatial control. Physical disruption of the BBB, on the other hand, is emerging as a promising method of delivering drugs to specific targets with a higher degree of spatiotemporal control.

## Physical mechanisms

Physical techniques can alter BBB integrity by destabilising TJs in addition to chemically mediated or hyperosmotic approaches. In recent years, work has focused on how they can induce selective and reversible BBB permeability. Figure 1c–f depicts the schematic of the effects of physical stimulation on the BBB. During stimulation, the bonds between TJ and AJ proteins are disrupted and decoupled, resulting in temporary opening of the BBB, which recovers with time after stimulation has ended (Fig. 1c). The specific proteins that are disrupted, the timescale over which this occurs, and the nature of the renewal of these junctions is a function of the specific disruption mechanism employed. For optical methods like photodynamic therapy and laser interstitial thermotherapy (Fig. 1d), disruption of the BBB occurs by local heating. In electrical stimulation (Fig. 1e), either transcranial stimulation, with electrodes placed on the skull, or electroporation, using penetrating electrodes, electrical currents generate increased permeability. In mechanical stimulation, ultrasound often in combination with injected microbubbles opens the BBB mechanically (Fig. 1f). A list of some of the relevant physical characteristics of brain tissue is provided in Table 2, where these quantities provide insight into the practical application of each of these methods. Each mechanism is discussed in more detail in the following sections.

**Table 2:** Relevant physical values for BBB stimulation

Property	Value	Reference
Trans-endothelial electrical resistance (TEER)	1500–8000 Ω cm^2^	[59]
Density, brain tissue	1.03 g/cm^3^	[60]
Conductivity, brain tissue	0.258 S/m	[61]
Volume specific surface area, BBB	150–200 cm^2^/g tissue	[59]
Total surface area, BBB (in adults)	12–18 m^2^	[59]
Acoustic attenuation, brain tissue	0.8 dB/cm*MHz	[62]
Safe electric current density, brain tissue	0.06 mA/m^2^	[63]
Safe electric charge injection capacity	0.054 C/m^2^	[63]
Thermal conductivity, brain tissue	0.527W/mC	[64]
Thermal attenuation coefficient, brain tissue	35.2/cm	[64]
Diffusivity	1.3 10^–7^ m^2^/s	[64]

### Optical stimulation

Phototherapy is the use of light at specific wavelengths to treat a range of diseases in a range of disciplines including oncology, optometry and dermatology [65–67]. Two types of phototherapies exist, namely photodynamic therapy, which uses photoactivated molecules or photosensitising drugs to trigger photochemical reactions, and laser interstitial thermal therapy, which uses photothermal agents to selectively heat tissue [67]. In either method, illuminating a specific brain region causes the internalisation of junction molecules, locally opening the BBB. Without further stimulation, the BBB recovers over time and junction proteins seal the paracellular space. In contrast to other physical stimulation methods, which demonstrate an almost instantaneous opening and closing of the BBB, optical stimulation opens the BBB after exposure and can last up to 48 h [68].

#### Photodynamic therapy

**Fig. 2:**
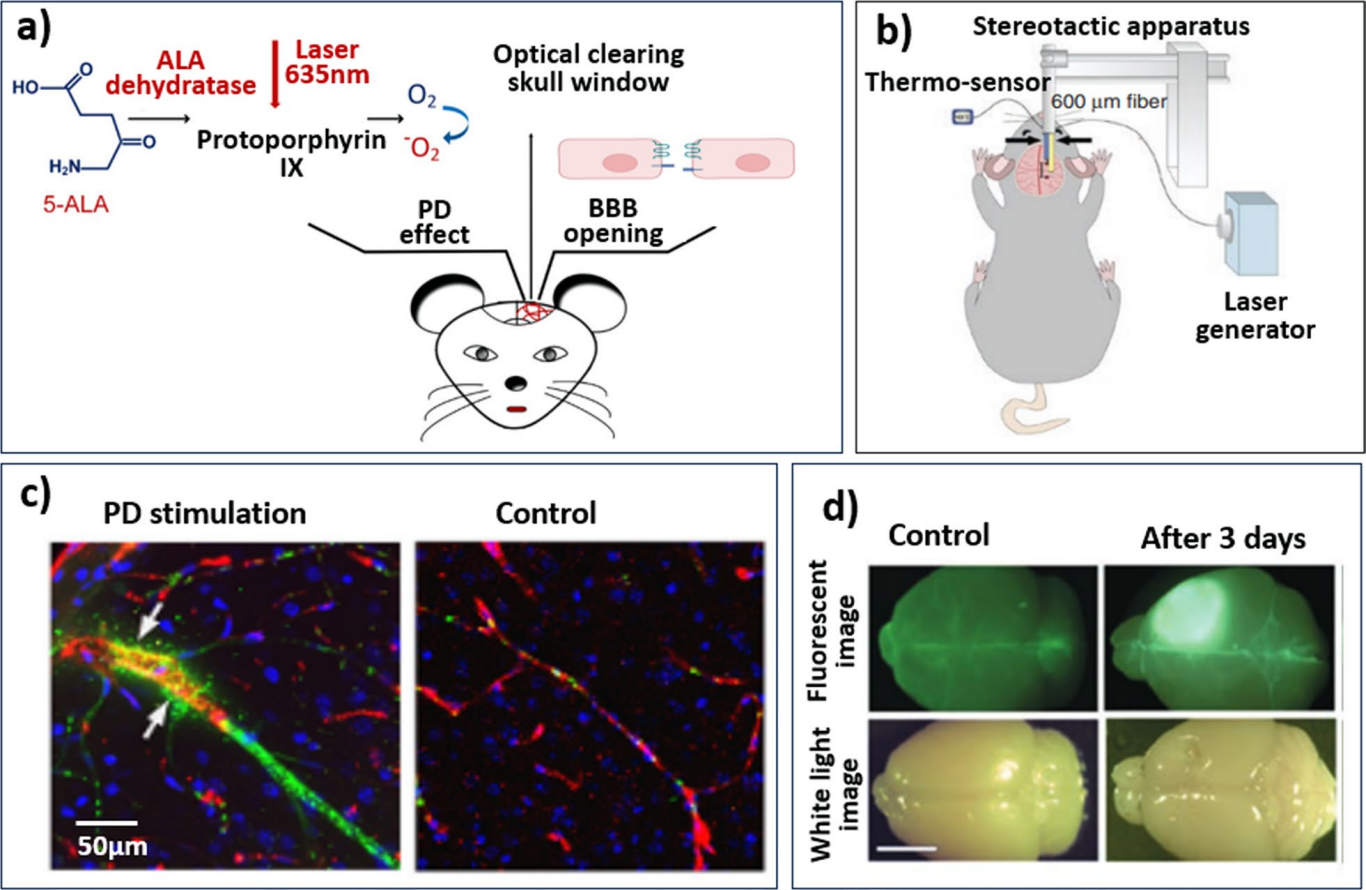
**a** Schematic of photodynamic therapy-induced BBB opening in an animal model, adapted from Zhang et al. [68]. 5-ALA, a photosensitising agent, is injected intravenously prior to light exposure. Through a small opening/window in the skull, light at an agent-specific wavelength (here 635 nm) is emitted into the brain leading to photochemical reactions and heating of the stimulated area. The thermal effect leads to the local opening of the BBB post-exposure. **b** Schematic depiction of the LITT delivery system in mice. The laser fibre (right arrow) is positioned 1 mm caudal to the thermo-sensor (left arrow). In contrast to PD, a photosensitising agent is not used and laser treatment is delivered via laser fibre, avoiding a skull window. Figure adapted from Salehi et al. [87] **c** experimental results of PD stimulation: confocal imaging of GM1-liposomes the with usage of markers of the neurovascular unit. Cell nuclei are labelled with DAPI, liposome leakage outside the vascular endothelial cells are labelled by antibodies. The white arrows show the sites of liposome leakage. Images were taken from Zhang et al. [68]. **d** LITT increases BBB and BTB permeability in vivo. Representative white light and fluorescence images of mouse brains harvested on the indicated days after intravenous fluorescein injection. LITT was performed in the right somatosensory cortex. Control = unmanipulated brain. Scale bar = 5 mm, taken from Salehi et. al. [87]

Photodynamic therapy (PDT) is a clinically approved anti-tumour treatment applied to a wide variety of cancers [69]. Figure 2a shows the schematic of PDT in a rat model. In order to activate a drug, a photosensitising agent or drug is administered intravenously to the target tissue, followed by illumination with light of a wavelength determined by the agent. Light activation triggers chemical reactions that interact with the tissue [65]. Light activation of photosensitisers (i.e. fluorescent dyes) generates reactive oxygen species which cause localised cell death via apoptosis, necrosis, and autophagy. In this method, light is delivered through an optical window in the skull [68]. PDT is currently applied to kill tumour cells, collapse tumour microvasculature, and to induce inflammatory responses that stimulate systemic immunity [70, 71].

Important physical parameters of PDT include the optical wavelength, the fluence (energy density, J/cm^2^) and the fluence rate (mW/cm^2^). Common photosensitisers include hematoporphyrin derivatives and 5-aminole-vulinic acid (ALA) [72, 73]. It is common to use fluences above 50 J/cm^2^ in clinical applications of PDT. There is, however, a tendency for these “high dose” regimes to result in oedema lasting longer than 3 days, which suggests more permanent damage to the vascular system as demonstrated in a rat model [74]. Multiple in vivo studies using mice and rat models, however, have also shown that PDT at fluences beneath the clinical standard can temporarily increase BBB permeability. For example, Zhang et al. demonstrated in vivo opening of the BBB to fluorescent liposomes using a wavelength of 635 nm (10–40 J/cm^2^, 40–100 mV, 250–400 s) in mice, as shown in Fig. 2c [68]. Additional rat model investigations demonstrated that even fluences on the order of 10 J/cm^2^ led to vasogenic oedema lasting up to 2 weeks [73, 75, 76]. More recently, Inglut et al. presented photodynamic priming as a low-dose (< 1.2 J/cm^2^) alternative, demonstrating a temporary increase in BBB permeability using human brain microvascular endothelial cells (HBMEC) in vitro, while maintaining high cell viability [77]. The transduction mechanisms of photodynamic therapy and photodynamic priming remain unconfirmed.

Comparisons across in vitro studies are complicated by differences in wavelength, fluence, fluence rate, photosensitive agent concentration, and cell type. However, given that a dose of 5 J/cm^2^ at 6 mW/cm^2^ reduced the viability of HBMEC by ~ 18% and mitochondrial activity by ~ 36% [77], the overall effect on BBB permeability in in vivo experiments is likely a combination of transient reorganisation of the cytoskeleton and long-term damage to the cell and mitochondria [68, 78]. The formation of actin stress fibres has been observed in this sub-cytotoxic regime, consistent with earlier in vitro studies on human umbilical vein endothelial cells (HUVECs) [79]. That study confirmed RhoA phosphorylation as the underlying mechanism for actin reorganisation, where RhoA protein plays a central role in regulating cell shape and polarity [80]. While the presence of RhoA hasn’t been confirmed in HBMECs directly, this would explain observed decreases in VE-cadherin expression following PDT [76], since RhoA inhibits VE-cadherin production. Photodynamic priming, however, did not significantly alter VE-cadherin expression, suggesting that this observation might be limited to higher fluences. Additionally, photodynamic effects induce microtubule depolymerisation in HUVECs [81]. Based on these effects, photodynamic priming, increases the circularity of HBMECs by 3–13% [77]. Aside from cytoskeletal morphology, Inglut et al. also observed changes in cell–cell junction morphology following photodynamic priming [77]. Adherens and tight junctions changed from a mature morphology in which VE-cadherin and ZO-1 were aligned continuously along the edge of the cell to an immature morphology in which they orient perpendicularly to the cell membrane. This agrees with previous studies into cancer cell transendothelial migration across HBMEC barriers, which also showed that TJ disruption, mediated by Rho/ROCK activation, led to increased transendothelial cancerous cell migration. This was due to TJ opening through rearrangement of the actin cytoskeleton and rendered TJ proteins such as occludin from insoluble to soluble state [82]. Since stress fibres anchor to cadherin complexes, the rearrangement of actin fibres does not explain the changes in VE-cadherin morphology [83, 84]. Instead, Zhang et al. suggest that β-arrestin1 might be responsible for VE-cadherin internalisation, as β-arrestin1 expression rose following PDT [76]. Indeed, Hebda et al. confirmed that phosphorylated VE-cadherin was internalised via interactions with β-arrestin1 and β-arrestin [85]. However, that study observed the effects of vascular endothelial growth factor on HUVECs; whether the same mechanism applies to HBMECs remains to be determined.

Overall, PDT-induced changes in junction morphology consistently increase BBB permeability to a variety of large molecules, regardless of the mechanism. The uniqueness of PDP lies in its spatiotemporal selectivity, which limits endothelial permeability to the site where light is applied. In addition to opening the BBB, the photodynamic effect provides a platform for developing treatments for brain diseases.

#### Laser interstitial thermo-therapy

Further light-based methods include laser interstitial thermo-therapy (LITT), used primarily in conjunction with magnetic resonance thermal imaging to ablate brain tumours [86]. Figure 2b depicts a schematic of LITT in an animal model. LITT uses laser energy to generate heat within the target tissue, which is delivered via a laser probe inserted directly into the tissue [86, 87]. LITT is a minimally invasive procedure used primarily in conjunction in magnetic resonance thermal imaging or CT scans, and it differs from PDT in that laser energy interacts directly with the tissue without the use of photosensitising drugs [87, 88]. The advantage of LITT lies in its precision and ability to precisely target the area of interest while minimising damage to the surrounding tissue. Clinical systems cause coagulative necrosis of tumour cells above 60 °C using either a 12 W, 1064 nm or 15 W, 980 nm laser. Sabel et al. showed hyperthermia in the peritumoral region (> 40 °C) disrupted the BBB in rodents [89]. Salehi et al. investigated LITT in vivo using mice (Fig. 2d), where BBB permeability, as measured by brain fluorescein accumulation, substantially increased within the first-week post-LITT, using 2 W power on continuous mode to deliver laser at 1064 nm through a fibre optic cable [87].

Many clinical studies assessing BBB permeability shed light on the full cellular mechanisms involved in hyperthermia-induced disruption. Leuthardt et al. demonstrated that LITT could be used to deliver the BBB-impermeant chemotherapy agent doxorubicin dosed intravenously at 200 mg/m^2^ into the brain. By monitoring the serum concentration of brain-specific enolase, they showed a peak increase in BBB permeability 1–2 weeks following LITT, with effects persisting up to 4–6 weeks. Enolase concentration rose from 175 ng/ml prior to LITT to 350 ng/ml between week 2 and 4 before falling again pre-exposure levels after week 6. [90]. Based on previous studies investigating the effects of LITT on nitric oxide levels in brain tissue, one theory proposes that LITT induces an increase in nitric oxide in the BBB. Nitric oxide plays an important role in regulating cerebral blood flow and metabolism, and its deficiency has been implicated in a variety of neurological and psychiatric disorders [91]. For example, Balanca et al. found that LITT increased nitric oxide levels in injured rat brain tissue, potentially contributing to a neuroprotective effect [92]. In addition, LITT may also contribute to transient increases in BBB permeability in vivo by causing heat shock proteins and nitric oxide secretion [93]. These findings suggest that LITT may be used as a potential therapeutic in conditions where nitric oxide is deficient, such as strokes and neurodegenerative diseases [94]. An in vivo study in rats, however, suggested that hyperthermic brain injury might be mediated by upregulation of nitric oxide synthase in neurons [95]. In summary, the relationship between hyperthermia and the upregulation of nitric oxide synthase is not yet fully understood, with the possibility that other circulating serum proteins produced by other organs may be involved.

When considering mechanisms for how LITT increases barrier permeability, lessons might be taken from studies of heat stress on endothelial cells [96]. Yamaguchi et al. used a combination of an in vivo mouse model and an in vitro pluripotent stem cell-derived model to study the effects of heat stress [96]. When cells were exposed to serum from mice exposed to heat stress, in vitro barrier permeability increased. When the in vitro cultures were exposed to heat stress, claudin-5 expression fell, PECAM-1 expression rose, and ZO-1 and occludin levels remained unchanged. Interestingly, expression of Pgp, an efflux transporter effective against multiple drug targets, also rose. This suggests a decrease in transcellular permeability, although this was not measured. According to Salehi et al., separate in vitro models showed an increase in endocytic vesicles following LITT [87]. However, they confirmed lower claudin-5 expression, albeit in mouse cells. Ultimately, current data suggested that LITT increases paracellular permeability by weakening tight junctions [66], although more research is needed to fully understand the mechanisms involved.

### Electrical stimulation

Electrical stimulation has been studied to treat Parkinson’s disease, epilepsy, paralysis and even psychiatric disorders. It seeks to modify inter-neural signalling by inducing or inhibiting action potentials with applied electrical potentials. A well-known example of electrical stimulation is deep brain stimulation, with application in the treatment of tremors in Parkinson’s disease [97, 98]. Recently, a study showed that patients’ quality of life improved even 15 years after intervention [63]. However, its efficacy in the treatment of neural dysregulation does not predict success in the use of electrical stimulation for treatment of other diseases. Electrical stimulation can be applied via either low-current transcranial or high-frequency penetrating electrodes to the brain. Depending on the method used, electric stimulation has a wide range of achievable spatial resolutions [19]. Transcranial stimulation, for instance, has been shown to modulate neurons within mouse brain regions of a few millimetres [99]. The neurovascular unit, however, is closer to 10 µm in diameter [100]. The spatial resolution is, therefore, relatively coarse compared to the size of the neurovascular unit. This means that while electric stimulation targets specific brain regions, it may not target specific cells within that region precisely [101, 102]. The BBB may also be affected differently depending on the electrical parameters and location of the stimulation.

#### Transcranial direct current stimulation (tDCS)

**Fig. 3:**
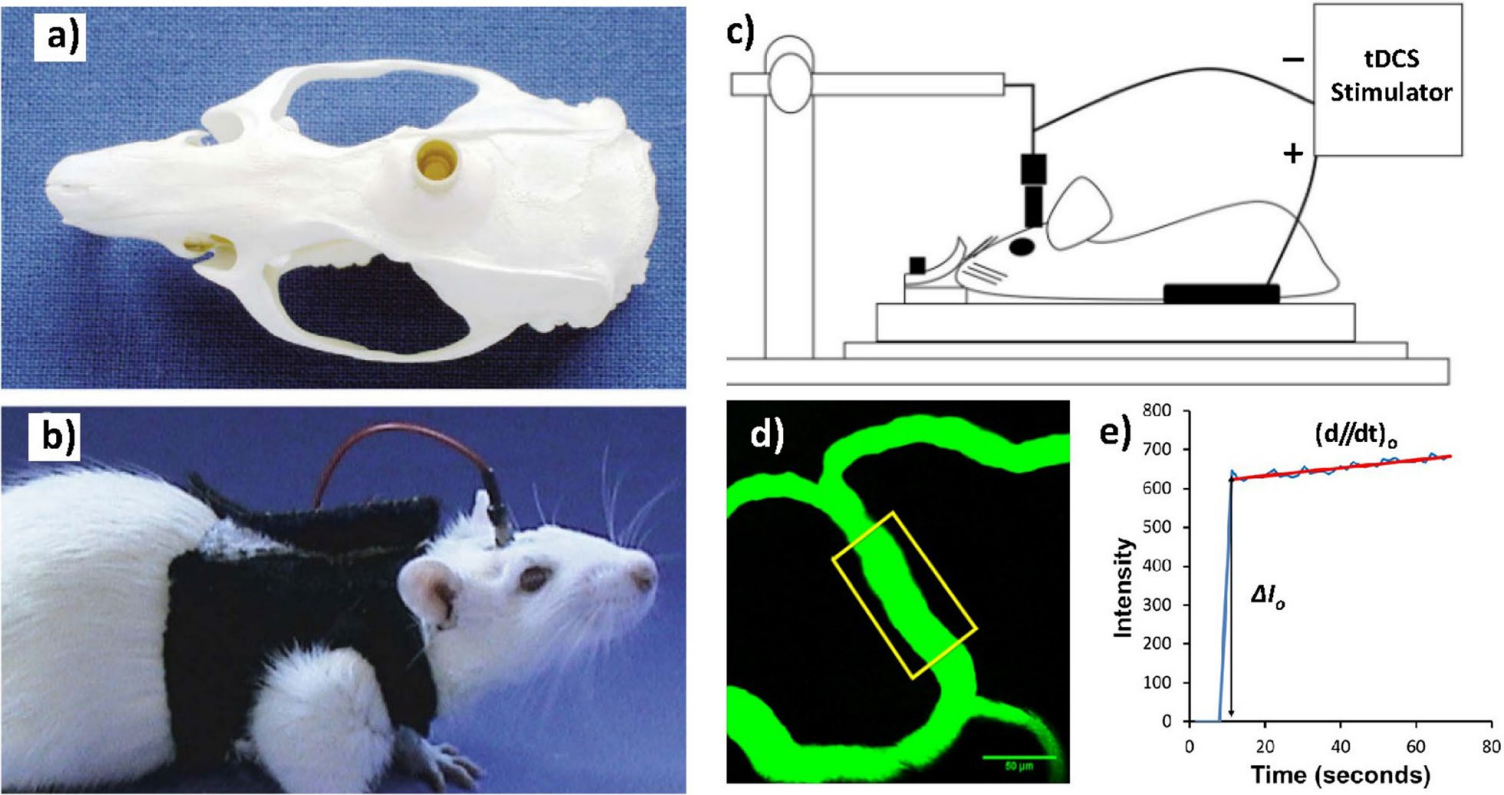
Electrode arrangement for transcranial DC stimulation in a rat. **a** The epicranial electrode (contact area = 3.5 mm^2^) is fixed onto the skull unilaterally above the frontal cortex (1.5 mm right and 2 mm anterior to bregma) using dental cement. **b** Before DC stimulation, the epicranial electrode is filled with saline solution. A large rubber plate mounted on the chest serves as the counter electrode, images adapted from Liebetanz et al. [104]. **c** Schematic of the setup for the tDCS. One electrode connects to the rat cranium and the counter electrode connects to the ventral thoracic region, **d** Determination of the BBB solute permeability with illustration of the scanning region of interest (ROI) comprising several microvessels. The yellow frame enclosed area is the ROI used to determine the BBB permeability to a solute. **e** Total fluorescence intensity in the ROI as a function of the perfusion time. Fluorescence intensity in the figure is proportional to the total amount of solute accumulating in the region surrounding the microvessel. Figure taken from Shin et al. [101]

Non-invasive transcranial direct current stimulation (tDCS) can be used to treat neurological disorders [103]. By placing electrodes on the scalp, a low electric current (in the mA range) can safely be delivered to modify neuroplasticity across wide regions of the brain. Figure 3a, b show electrode arrangements used for transcranial DC stimulation in a rat [104], where one electrode connects to the rat cranium and the counter electrode connects to the ventral thoracic region (Fig. 3c) [101]. In a rat model, Liebetanz et al. showed that lesions did not develop until a current density of 142.9 A/m^2^ or an estimated cumulative charge density of 52400  C/m^2^ was applied [104]. Subsequent clinical and laboratory studies have remained far below this limit. For example, Marceglia et al. applied 1.5 mA for 15 min to patients with Alzheimer’s disease, yielding 0.06 mA/cm^2^ of current density and 0.054  C/cm^2^ of cumulative charge density [63]. A working memory test showed that tDCS modulated both high-frequency and low-frequency cortical oscillations. Within the context of its original clinical intent, tDCS has demonstrated benefit for decades and has been relatively well characterised.

Rather than studying tDCS's neuromodulatory effects, recent studies have focused on its effects on the neurovascular unit. Cancel et al. presented a modified Transwell-based setup which allowed them to apply a 1 mA electric current to a monolayer of bEnd.3 mouse brain endothelial cells under either pressure-driven (i.e. convective) or diffusive flow. Under both flow circumstances, applying an electric current significantly increased the permeability of the barrier to a large molecular weight colloid (TAMRA-modified dextran, 70 kDa) but not to a low molecular weight fluorescent marker (TAMRA, 430 Da). Given that the transport of the smaller solute was dominated by diffusion and that its permeability was therefore not affected by the application of electric current, the authors surmised that the increased permeability to the larger solute was caused by increased convection through the gaps between TJs. If the electric current had caused widening of the gaps or an increase in the number of gaps, the permeability of the smaller solutes would also have been affected [105]. Xia et al. showed tDCS downregulated and delocalised ZO-1 in bEnd.3 monolayers [106], whereas Cancel et al. showed no such TJ damage [105].

In vivo studies have, however, revealed additional effects. Rather than immediately returning to normal, Shin et al. observed increases in membrane permeability that lasted approx. 20 min following tDCS treatment of 0.1–1.0 mA for 20 min in a rat model [101] (Fig. 3d). The BBB permeability to sodium fluorescein (376 Da) also increased, suggesting another mechanism in addition to increased convection through TJ.

When it comes to the effects of tDCS on endothelial cells, numerous studies paint a multi-faceted range of molecular targets. As a result of applying a nitric oxide synthase inhibitor, Shin et al. found that permeability effects were sharply reduced, suggesting that NO-mediated opening may also contribute to the permeability effects in vivo in rats [101]. Later studies from the same group confirmed this dependency in in vitro human and rat cell monolayers [101]. With tDCS, the interplay between processes that results in this increased nitric oxide production is unclear, but calcium has been offered as one potential mediator. For example, Monai et al. showed tDCS induced release of Ca^2+^ from mouse astrocytes in vivo [99] and Trivedi et al. found that low-frequency (0.5–2.0 Hz) electric fields amplified nitric oxide synthesis in bovine aortic ECs in vitro in the presence of extracellular Ca^2+^ [91]. Regardless, Trivedi et al. also observed an apparently Ca^2+^-independent pathway for nitric oxide synthesis when cells were exposed to these electric fields (albeit alternating rather than direct). Likewise, Tarbell et al. reported that flow increased through intercellular junctions [107], indicating a possible connection between Cancel et al. s’ convective effects and others’ in vivo effects [101, 106, 108]. Aside from nitric oxide, Bai et al. showed small DC electric fields applied to HUVECs upregulated VEGF [109], which increases brain microvascular permeability [110]. Additionally, tDCS transiently degraded endothelial glycocalyx and ECM; heparan sulphate and hyaluronic acid were downregulated. In the same study, tDCS eliminated the permeability difference between positively and negatively charged solutes of comparable size in vivo. Xia et al. suggested this effect previously, where tDCS raised the permeability of bovine serum albumin (~ 69 kDa with a negative charge) above that of 70 kDa dextran in vitro, even though it had been lowering at baseline [106]. It is interesting to note that tDCS only had an effect on the BBB itself; once the molecules reached the ECM of brain tissue, there was no change in effective diffusivity.

While tDCS has been shown to have various cognitive and therapeutic effects, further research is needed to determine the safety and efficacy of this approach as well as the potential risk of increased exposure to toxins and pathogens [111, 112]. In addition, different operating requirements of the probes can have different effects on the blood–brain barrier [104, 111]. Lastly, the probes for electroporation are highly invasive. It further remains to be seen if these can be inserted into other deep brain regions without causing long-term damage.

#### Pulsed electric fields

In contrast to transcranial Direct Current Stimulation, pulsed electric field methods intermittently stimulate the brain. For the sake of clarity, here we use the classification of “pulsed electric field methods” to refer to a range of clinical applications such as electrochemotherapy, tumour treating fields [113], non-thermal irreversible electroporation (NTIRE) [61, 114] low pulsed electric field [115] deep brain stimulation [116] or high-frequency irreversible electroporation (HFIRE) [117, 118]. While these techniques have distinct clinical purposes and effects, they differ largely in terms of certain key process parameters, such as pulse amplitude, pulse duration, pulse application frequency, field polarity, and therapy duration (or number of pulses). For example, NTIRE and HFIRE ablate tumours by stimulating with pulse amplitudes typically exceeding 500 V/cm, creating irreversible pore structures in the cell membrane to kill cells [114, 117]. Early MRI outlined how the effects within the tissue correlated with the field strength at that point, depending on the position of the stimulating electrodes and the electrical properties of the tissue [61, 114]. Outside the high field strength regions, lower amplitude fields (< 500 V/cm) cause reversible electroporation, allowing molecules to permeate the BBB through a transcellular pathway. With even lower field strengths (< 140 V/cm), Sharabi et al. have demonstrated both in vivo and in vitro methods for reversibly disrupting the BBB via the paracellular pathway, without inducing electroporation effects [115]. In that low pulse electric field approach, 10 pulses applied for a duration of 50 μs each at a rate of 1 pulse/s could create the reversible, non-electroporative effect. Interestingly, Bonakdar et al. induced similarly reversible increases in paracellular permeability using an in vitro model to analyse deep brain stimulation pulses [116]. There, low amplitude (25 V/cm) pulses were applied for a shorter pulse duration (10 μs) at a much higher frequency (200 pulses/s) for 30 min, increasing the permeability of an endothelial monolayer to both high- and low-molecular weight tracers. In total, electrical stimulation methods offer a range of clinical possibilities, including the ability to simultaneously ablate tissue regions and reversibly disrupt the BBB in the surrounding tissue for more effective drug delivery strategies.

The transduction mechanisms of pulsed electric field methods remain unconfirmed, though in vivo experiments are beginning to paint a clearer picture than previous in vitro investigations have. A recent study from Partridge et al. showed that BBB disruption caused by HFIRE was mediated largely by post-translational modification of claudin-5 and occludin [118]. Ubiquitination of both tight junction proteins was associated with reduced protein expression via molecular recycling as well as delocalisation from the cell membrane to the cytosol, resulting in reduced tight junction stability. These results pair well with a study from Salvador et al. that attributed the reversible disruption of BBB permeability with tumour treating fields to phosphorylation of claudin-5 [113]. That study also showed delocalisation of claudin-5 over the course of 72 h following treatment. Combined with earlier in vitro studies that did not show substantial changes expression of other junction proteins like VE-cadherin and ZO-1 [115], the literature seems to suggest that this non-electroporative disruption is mediated by modification of existing cellular structures, rather than broad level protein expression. The dynamics of post-translational modification could also underlie the reversible nature of changes in permeability. Comparing the results from the in vitro and in vivo studies also demonstrates the advantage of animal models in identifying these changes between experimental conditions. Due to varying expression profiles of different primary or immortalised cell lines under in vitro conditions, it can be difficult to identify which mechanisms play how large of a role in the natural environment.

#### Irreversible and reversible electroporation

**Fig. 4:**
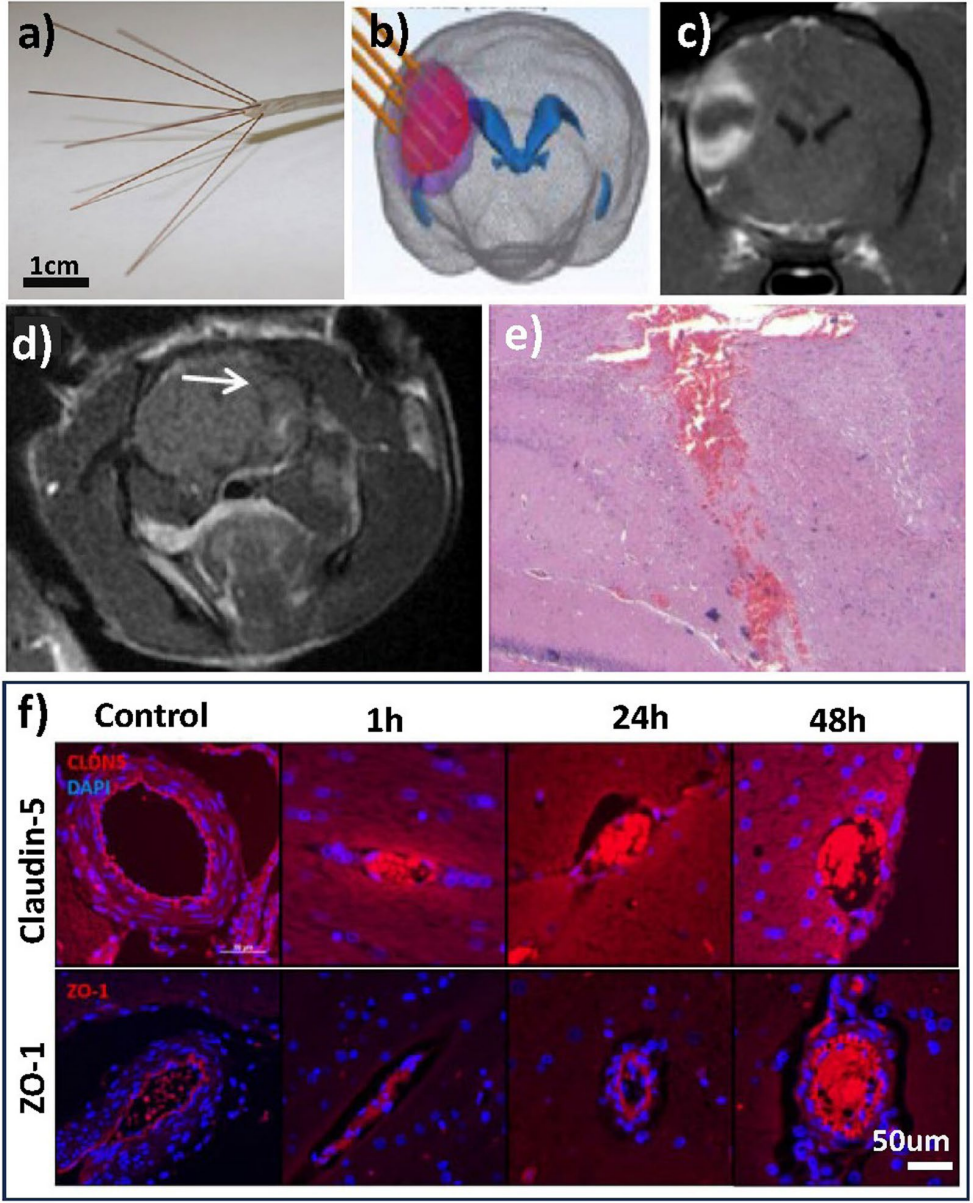
HFIRE stimulation, where **a** illustrates the distal end of the CETCS system with microneedles arborized from the primary cannula in convection-enhanced delivery CED catheters. **b** Schematic needle insertion in HFIRE induced BBB disruption and **c** post-contrast MRI images after gadolinium enhancement surrounding tumour area after treatment. Figures taken from Partridge et al. [119]. **d** MRI analysis of control brains in electroporation treatment with visible needle path (white arrow) obtained 30 min post treatment and **e** Histopathology of brain region with minimal bleeding along electrode insertion path. Figures taken from Sharabi et al. [61]. Immunofluorescent staining of transverse brain samples following intracranial HFIRE revealed a deceased in claudin-5 and ZO-1 reactivity 1 h post-treatment followed by gradual increase over time compared to control. Scale bar is 50 µm across all images. Image taken from Partridge et al. [118]

The main medical application of electroporation is to eliminate tumours by delivering high voltage short pulsed electric fields (10–100 µs) through minimally invasive electrodes directly to the target tissue. Cellular response varies depending on the strength of the applied electric field. Irreversible electroporation uses high electric fields (> 500 V/cm) leading to irreversible membrane permeabilisation and subsequent cell death, a common method in Glioblastoma tumour ablation [118, 119]. Here, an intracranial needle electrode is placed in the target tissue and the outer surface electrode placed on the skin or skull. Figure 4a depicts such distal end of microneedles, which are inserted into the brain (Fig. 4b), leading to enhanced electroporation in the surrounding tissue (Fig. 4c) [119]*.* The strongest electric field is found surrounding the electrode region, causing irreversible electroporation. This electrical field gradually weakens with distance resulting in a reversible electroporation effect in peripheral regions [120]. While these microneedles leave a visible insertion path (Fig. 4d) they only cause minor bleeding without further damage (Fig. 4e). Reversible Electroporation uses low field strengths (< 500 V/cm) resulting in a reversible cell membrane permeabilisation. This method is often applied to targeted delivery of molecules by forming temporary, reversible channels in the cell membrane [120]. Applied to the BBB, controlled electrical pulses create temporary and reversible pores in the endothelial cells. These pores allow for the passage of therapeutic agents or drugs that would typically be unable to cross the BBB [117, 118]. In vivo studies in rat model by Hjouj et al. and Sharabi et al. demonstrated that applied electric fields between 200 and 600 V/cm, using 90 50 µs pulses at 1 Hz result in successful BBB disruption with limited damage to surrounding tissue [61, 114]. At low voltages, HFIRE is an effective intracranial combination therapy for facilitating drug diffusion into brain parenchyma below lethal levels of energy ensuring reversible BBB opening [117]. Several in vivo studies have examined BBB disruption as a function of pulse number, where a higher number of pulses requires lower powers to achieve paracellular passage [115–117, 121]. Partridge et al. and Lorenzo et al. investigated temporal BBB disruption through high electric fields with 600 V/cm in rat models, demonstrating disruption for 1–72 h and an intact BBB at t = 96 h post-treatment, though tissue damage may result at the electrode insertion area [117, 118]. A further analysis of the proteins and cytoskeleton found that F/G-actin ratios and TJ proteins concentrations decreased, and that TJ protein ubiquitination increased within 1–48 h after treatment. Additionally, cytoskeletal and TJ protein expression recovered to pretreatment levels by 72–96 h, with claudin-5 and ZO-1 expression increasing [118] (Fig. 4f). Therefore, cytoskeletal remodelling and altered regulation of TJ proteins causes transient disruption of TJ complexes, leading to the opening of the BBB.

#### Low-pulsed electrical fields (L-PEF)

For non-invasive drug delivery, L-PEFs are a new and promising approach to increase BBB permeability selectively and temporarily. Voltage pulses, far below the electric-field threshold for electroporation-induced BBB disruption set at about 500–700 V/cm, are applied to the skull via non-penetrating electrodes [122]. The resulting electrical fields destabilise the cell membrane and induce nano-scale pores in the cell’s membranes [122]. L-PEF can be seen between reversible electroporation, applying extremely low voltages [61, 121], and tDCS, using extracranial electrodes at similar current densities but pulsing the signal compared to consistent current ramped up and down [105]. Operating at lower intensities reduces the risk of cellular damage and other side effects. Thus, L-PEF induces temporary and reversible opening of the BBB opening, allowing for precise control over drug delivery while minimising potential harm to brain tissue [123]. For example, Sharabi et al. demonstrated increased paracellular barrier leakage in vitro*,* using human BLEC. By applying low voltage pulses of down to 10 V and pulse duration of 90–400 μs, they detected an up to 40% increase of NaF permeability and decrease in TEER [115]. Also, Rajagopalan et al. investigated reversible BBB disruption in vitro using primary HUVEC and in vivo using rat model [123]. Both in vitro and in vivo, pulses with electrical fields of 1000 V/cm induced significant alterations to the BBB. In vitro, pulses of 1000 V/cm led to actin remodelling and tight junction disruption and internalisation but also reduced cell viability. In animal models, higher energy delivered corresponded with a larger region of the brain undergoing BBB disruption peaking at 1500 V, 100 μs and 100 pulses at 10 Hz. Furthermore, recent studies demonstrate effective and safe BBB disruption in mice models. In an MRI study, Sharabi et al. detected a linear increase in area of BBB disruption with applied voltage ranging 0–300 V at 100 pulses, peaking in a 3–3.2 times larger area without signs of oedema, damage or bleeding [122]. Cooper et al. demonstrated efficient doxorubicin delivery in vivo, where treatment with 100 pulses at 200 V led to a 29-fold higher BBB opening in mice brains, resulting in concentrations of 0.5 μg Doxo/gr brain compared to 0.03 Doxo/gr brain for the control group [124]. While these results demonstrate that PEFs increase the permeability of the paracellular-endothelial route, the underlying mechanism are still yet to be determined.

### Mechanical stimulation

**Fig. 5:**
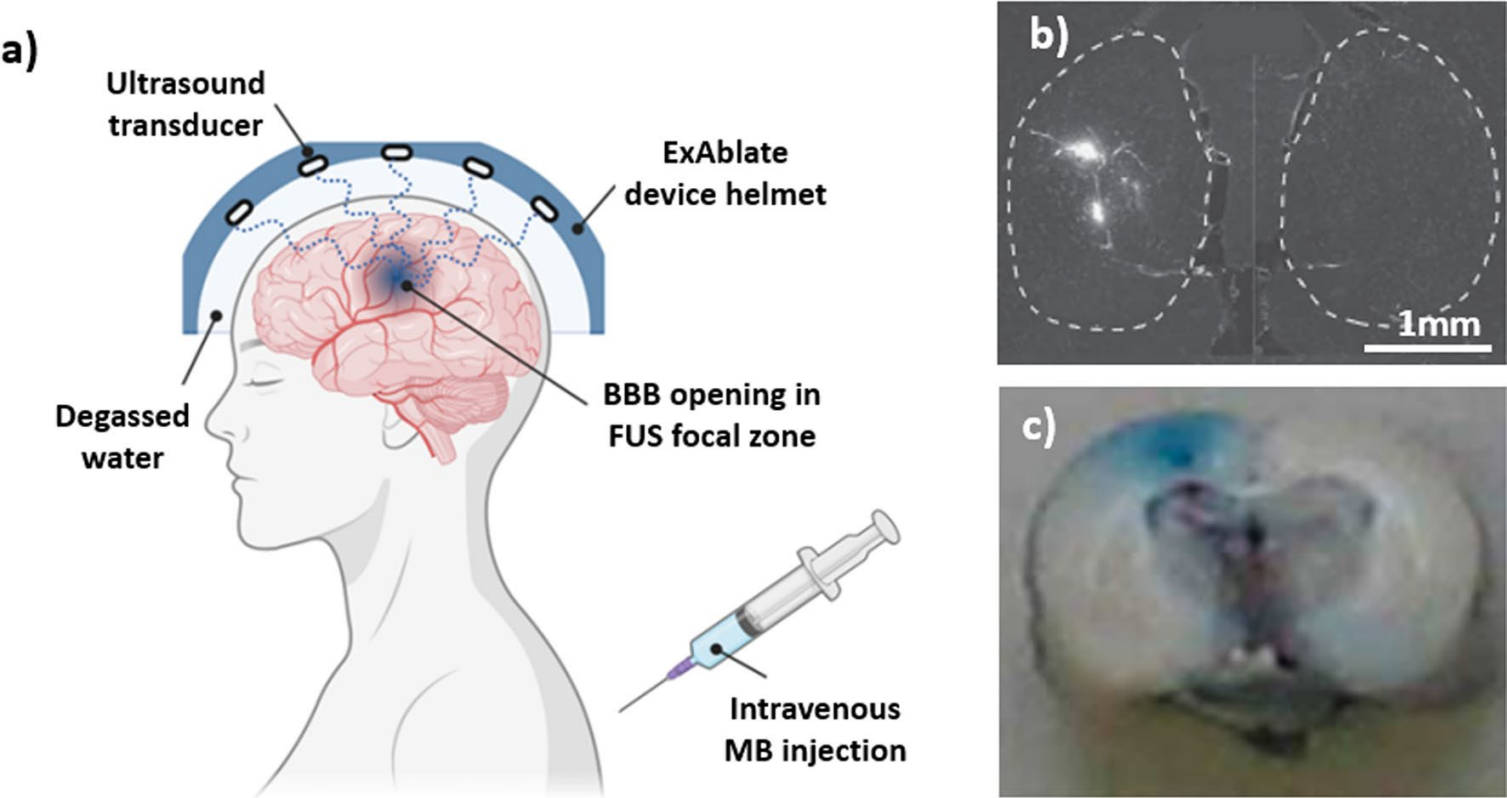
Schematic representation of blood–brain barrier opening in Alzheimer’s disease patient in vivo and patient-derived model in vitro. **a** Schematic of a magnetic resonance (MR)-guided ExAblate device used in the first successful blood–brain barrier (BBB) opening in Alzheimer’s disease (AD) patients. System consists of a hemispherical helmet lined with > 1000 independent transducer elements delivering low frequency ultrasound treatment to the prescribed target. The helmet is positioned in the specialised MRI bed with stereotaxic frame and the space between patient’s head and the helmet filled with degassed water for acoustic coupling. Microbubble (MB) administration is carried out using repeated bolus injection or a continuous infusion. Reversible BBB opening occurs in the defined ultrasound focal zone. Figure taken from [161]. **b** fluorescence images of FUS treated at US pressure of 0.84 MPa mouse brains (left side) compared to non-treated (right side), each injected with 500 kDa size dextran’s. Dashed lines indicated boundaries of the hippocampal regions. Figure taken from Chen et al. [142]. **c** Detection of BBB opening in rat brain, with left hemisphere sonicated at an intracranial pressure amplitude of 1.25 MPa in the presence of microbubbles at a dosage of 0.1 mL/kg and no treatment to the right side. Macroscopic inspection of 2–3 mm thick coronal section (front side of the brain) showed penetration of Evans Blue (molecular weight of 960.8 Da) into the underlying cortex. Figure taken from Alonso et al. [143]

Ultrasound disrupts the BBB through mechanical means. In clinical trials, low-intensity pulsed ultrasound (LIPU) [125] and focused ultrasound (FUS) [126] have both been shown to disrupt the BBB safely and effectively. In LIPU, a transducer is implanted through the skull to direct waves to a preselected region [127]. In FUS, however, waves are transmitted through the skull using various external transducers. Additionally, magnetic resonance imaging is often used for target planning and thermal monitoring, leading to techniques such as magnetic resonance-guided focused ultrasound (MRgFUS) [128]. To reduce the acoustic power required to open the BBB, microbubbles are injected systemically prior to US treatment [129]. When acoustic waves are present, these microbubbles oscillate, transmitting mechanical forces to the surrounding vessel walls as they flow through the vasculature [129]. A schematic of the application is depicted in Fig. 5a. This allows more targeted disruption through mechanical mechanisms [130], in contrast to other clinical uses of high-power US which employ thermal effects for tissue ablation [131].

Despite LIPU's ability to overcome the skull's reflection and distortion of ultrasound beams, FUS has been studied more extensively because of its relative non-invasiveness. In fact, FUS is approved by the FDA for the symptomatic treatment of Parkinson’s tremor [132], and safety and feasibility studies have investigated FUS in the context of glioblastoma [133, 134], amyotrophic lateral sclerosis (ALS) [135], Alzheimer’s, as well as several neuropsychiatric disorders [136]. As an example, Mainprize et al. found that MRgFUS treatment with 4–15W sonication power enhanced T1-weighted MRI contrast by 15–50% for up to 24 h, as well as increased chemotherapeutic delivery to tumours and peritumoral tissues [134]. Abrahao et al. saw similar results for ALS patients, including the resolution of BBB opening within 24 h, and they also reported no temperature elevation during MRgFUS [135]. Further, Lipsman et al. demonstrated that repeated openings of the BBB were well tolerated by Alzheimer’s patients, using MRgFUS twice in the frontal lobe [130]. While these studies were small in scope and patient number, they offer promising indications for FUS’ usefulness going forward. Current drawbacks of this technique are the limited tissue penetration, the long treatment procedure, safety, and cost efficacy. Lastly, MRgFUS is not suitable for patients with certain medical conditions or body habitus.

When investigating the effectiveness of US techniques for BBB disruption, many parameters can be adjusted. The most common reported device controls include acoustic frequency, sonication duration, sonication power, burst length, and burst repetition frequency. Aside from the device itself, microbubble properties such as concentration, size distribution, and circulation time, as well as tissue properties like vascular density, also affect the degree of BBB opening. Here, animal models are helpful for exploring the parameter space [129, 137–146]. Typical transducer frequencies include 0.5, 1.0, and 1.5 MHz, with peak negative pressure amplitudes in situ between 0.3 and 1.0 MPa. Burst length and burst repetition frequency typically range from 1 to 100 ms and 1 to 5 Hz, respectively, with sonication durations of 20 s to 11 min. Chen et al. showed in a mice model that the degree of BBB opening depends on the amplitude in a setup with 1.5 MHz transducer frequency, 1.3 ms pulse duration, repetition frequency of 5 Hz, and 11 min sonication duration [142]. At these settings, 0.51 MPa is sufficient to enhance the delivery of agents < 70 kDa in mice, whereas 0.31 MPa was only capable of delivering agents < 3 kDa. Figure 5b shows diffused dextran of size 500 kDa under FUS exposure compared to a control. Microbubble cavitation is stable at low powers, but above a threshold of approximately 0.8 MPa, inertial cavitation causes the bubbles to burst, damaging surrounding vessels in both human and animal models [127, 130]. During stable cavitation, though, the degree of BBB opening tends to increase with increasing microbubble diameter [147–149]. For more information on the effects of FUS parameters on BBB opening, the reader is referred to Aryal et al. [150].

FUS treatment increases BBB permeability via both transcellular and paracellular pathways. Early studies by Sheikov et al. showed increased vesicular formation, fenestration and channel opening in addition to the widening of tight junctions in brain microvascular cells in vitro 1–2 h after sonication [129, 146], where the use of immunostained ZO-1, claudin-1, claudin-5, and occludin confirmed that TJs had been disrupted [146]. Expression of occludin, claudin-5, and ZO-1 fell more than 50% immediately following sonication, and ZO-1 delocalised away from TJs. Approximately 24 h after sonication, immunosignals returned to control levels, indicating that BBB disruption had been fully reversed. Alonso et al. hypothesized that the delocalisation of ZO-1 might explain their observations of increased gap junction plaque size in astrocytes and neurons [143]. They showed that two key gap junction proteins, connexin36 and connexin43, reorganised into larger junctional plaques following FUS treatment, again in a reversible manner demonstrated in vivo rat model. Since ZO-1 regulates gap junction size under normal conditions, the removal of ZO-1 from the cell membrane might explain the increased gap junction size, possibly as part of a neuroprotective response. Figure 5c shows leakage of blue Evans through BBB opening at an intracranial pressure amplitude of 1.25 MPa in the presence of microbubbles at a dosage of 0.1 mL/kg.

FUS disrupts the BBB by increasing shear stress of ECs, but the exact signalling cascade is unknown [151]. Numerous studies have shown the effects of shear stress on regulating endothelial phenotype in vitro [152]. Shear stress dependent potassium channels, for example, hyperpolarise the cell membrane, allowing endothelial cells to vasodilate in response to increased shear via Ca^2+^-dependent activation of nitric oxide synthase [153]. Additionally, VE-cadherin mediates a shear response by modulating TJs through multiple pathways [154, 155]. Tzima et al. reported on a mechanosensory complex consisting of PECAM-1, VE-cadherin, and VEGF-R2 that acts as an upstream activator of multiple pathways via PI3K activation in vitro [156]. Jalali et al. found in a rat study that FUS treatment increased phosphorylation of protein kinase B (Akt), confirming that the PI3K/Akt pathway disrupts the BBB [137]. However, Akt phosphorylation remained high after TJ stability had returned, implying that, additional mechanisms must be responsible for restoring BBB stability if Pi3K/Akt signalling was caused by BBB disruption.

### Comparison of different stimulation methods

In terms of their mechanisms, target specificity, and safety considerations, each of optical/thermal, electric, and ultrasound-based/mechanical stimulation has its advantages and challenges. The key effects and, where known, mechanisms of each method are summarised in Table 3. Ultimately, any physical disruption method that can successfully impact paracellular transport mechanisms via TJ modification has the potential to impact cell function in other ways. The added energies can result in deleterious effects on cell viability at sufficiently high exposure doses, therefore the key operating parameters and appropriate ranges for different methods are summarised in Table 4.

**Table 3:** Mechanisms in stimulated BBB opening

Method	Effect/underlying mechanism	Ref
PDT	• RhoA phosphorylation leads decreased VE-cadherin expression • VE-cadherin and ZO-1 orientation shift perpendicular to cell membrane (immature state) • Actin rendering, occludin from insoluble to soluble state	[80] [76] [82]
LITT	• Heat stress TJ proteins, increase in NO, Clauding-5 expression decreases	[157]
tDSC	• Increases convection through TJs • Downregulation of ZO-1 and nitric oxide mediated opening	[99] [106]
L-PEF	• Remodelling of Actin, decrease in VE-cadherin and ZO-1 expression	[115, 123]
EP	• F/G-actin ratio decrease, TJ proteins decrease, actin remodelling	[121]
FUS	• Reduced expression of occludin, claudin-5, and ZO1 • Shear stress-dependent CA^2+^ channels, hyperpolarise cell membrane causing vasodilation • Nitric oxide synthase activation, VE-cadherin shift (immature state)	[129, 153] [152]

**Table 4:** Representative reported stimulation parameters

Parameter	Value	Ref
Optical: Thermal energy density brain tissue in PDT	50 J/cm^2^	[68]
Electrical: Reversible cell membrane permeabilisation	500 V/cm	[120]
Electrical: Applied electric field maximum	500 V/cm	[120]
Electrical: Voltage to distance ratio for BBB opening	200–400 V/cm	[122–125]
Threshold electroporation	400–600 V/cm	[158]
FUS: Safe and effective sonication power	4–15W	[144]
FUS: Effective BBB frequency range	0.5–1.5 MHz	[144, 145]
FUS: Effective acoustic pressure	0.3–1.0 MPa	[127, 144]
FUS: Effective pulse duration	1.3 MHz	[127, 145]
FUS: Microbubble bursting	> 0.8 MPa	[136, 144]

Optical or thermal stimulation relies on using light or heat to trigger specific molecules or nanoparticles in the BBB. This activation can lead to temporary BBB permeability for drug delivery. We distinguish between the two main options, Laser interstitial thermotherapy LITT and Photodynamic therapy PDT, where LITT uses laser energy directed to generate heat directly while PDT uses a photosensitizing agent and light to produce heat. When using targeted light sources or nanoparticles, both methods offer excellent spatial precision, including in deep brain regions. These methods have shown some advantages in terms of safety since they are relatively non-invasive. However, concerns about potential tissue damage due to heat and long-term effects of light exposure require careful evaluation.

Electric stimulation involves applying electrical currents directly to the brain or targeted brain regions using techniques like transcranial direct current stimulation (tDCS), irreversible and reversible electroporation (EP) and low-pulse electrical fields (L-PEF). The key difference is the non-invasiveness of tDCS and L-PEF with electrodes only placed on the skull, compared to electroporation, which requires brain-tissue-penetrating electrodes. The electrical currents alter the membrane permeability of BBB endothelial cells temporarily. It can be challenging to achieve precise targeting using electric stimulation, as electric current spreads unevenly throughout the brain tissues. There may also be some safety risks associated with electric stimulation since high currents or prolonged stimulation periods could cause unwanted neural activation or tissue damage. Overall, electric stimulation offers a straightforward approach but lacks precision and can lead to long-term tissue damage in the case of implanted electrodes.

Mechanical or acoustic stimulation typically involves the use of focused ultrasound (FUS) combined with microbubbles. The microbubbles oscillate under the influence of ultrasound, creating transient disruptions in the BBB and enhancing drug delivery. FUS provides the best spatial targeting and control of all physical methods, allowing precise control over the area of BBB opening. Acoustic stimulation has shown promise for BBB opening with a relatively good safety profile, and its efficacy has been confirmed in several clinical studies. However, concerns about potential side effects like microhemorrhages and inflammation require further investigation.

#### Current state of clinical applications

While safety and efficacy studies in animal models have shown promising results for physical stimulation methods, clinical studies demonstrating BBB opening to increase drug delivery are still in an early state. LITT and FUS lead the race towards therapeutic applications. Investigating LITT, Leuthardt et al. conducted a pilot clinical trial between 2013 and 2018, studying magnetic resonance imaging (MRI)-guided laser surgery (MLA) and doxorubicin hydrochloride in treating 14 patients with recurrent glioblastoma multiforme between, demonstrating increased BBB permeability [90]. FUS was approved in 2019 by the US FDA for clinical trials in 27 recurrent glioblastoma patients to deliver the chemotherapy drug carboplatin, with results still pending [159]. In a pilot study in 2018, Lipsman et al. demonstrated safe, reversible, and repeated opening of the BBB using MRgFUS in 5 patients suffering from Alzheimer’s Disease. Furthermore, cognitive scores didn’t decline within 3 months post treatment [130]. According to *clinicaltrials.gov*, Clinical trials investigating therapeutical applications of FUS are ongoing with currently 22 registered, with 7 active and 14 recruiting [160]. This growing body of evidence suggests that BBB modulation holds great potential for revolutionising the treatment of neurological diseases.

## Conclusion

The delivery of therapeutic agents to the brain can be modulated by physical disruption approaches based on optical, electrical and mechanical stimulation. While chemical methods to circumvent the BBB have been widely studied, clinical limitations include high failure rates and non-selective tissue effects. Physical methods, in contrast, offer the potential for spatiotemporally controlled modulation of the BBB, allowing temporary and reversible opening. The mechanisms of these physical methods have been a topic of recent examination, with available evidence suggesting that they can selectively disrupt tight junctions and increase paracellular transport. Focused ultrasound in particular has emerged as a leading clinically applied technique due to its ability to non-invasively achieve localised BBB modulation with minimal damage to surrounding tissue. Further research, however, is still required to determine the signalling cascades responsible for barrier disruption for each technique on a mechanistic level. Another key question is how BBB stability is restored following the disruption to ensure these methods can be applied without causing adverse effects, such as tissue damage, inflammation, or long-term alterations in BBB integrity. It may be possible to exploit mechanisms discovered here to develop therapeutic windows that could be activated for a defined amount of time, possibly mitigating the off-target effects of the techniques currently being studied. On a disease level, understanding the BBB’s role in early-stage pathology is a key hurdle to designing appropriate therapeutic strategies. On a system level, understanding how the BBB interacts with the remaining components of the neurovascular unit might uncover improved methods of diagnosing and preventing disease.

## Data Availability

Not applicable.

## Abbreviations

BBB: Blood–brain barrier
EC: Endothelial cell
TJ: Tight junction
AJ: Adherens junction
HBMEC: Human brain endothelial cells
HUVEC: Human umbilical vein endothelial cells
PDT: Photodynamic therapy
LITT: Laser interstitial thermo therapy
tDCS: Transcranial direct current stimulation
HFIRE: High frequency irreversible electroporation
L-PEF: Low pulse electrical fields
LIPU: Low intensity pulsed ultrasound
FUS: Focused ultrasound
MRgFUS: Magnetic resonance guided focused ultrasound

## References

[1] Abbott NJ (2000). Inflammatory mediators and modulation of blood-brain barrier permeability. Cell Mol Neurobiol.

[2] Daneman R, Prat A (2015). The blood-brain barrier. Cold Spring Harb Perspect Biol.

[3] Ballabh P, Braun A, Nedergaard M (2004). The blood—brain barrier: an overview structure, regulation, and clinical implications. Neurobiol Dis.

[4] Nelson AR, Sweeney MD, Sagare AP, Zlokovic BV (2016). Neurovascular dysfunction and neurodegeneration in dementia and Alzheimer’s disease. Biochim Et Biophys Acta (BBA)—Mol Basis Dis.

[5] Zhao Z, Nelson AR, Betsholtz C, Zlokovic BV (2015). Perspective establishment and dysfunction of the blood-brain barrier. Cell.

[6] Erickson MA, Banks WA (2013). Blood-brain barrier dysfunction as a cause and consequence of Alzheimer’s disease. J Cereb Blood Flow Metab.

[7] Hendricks BK, Cohen-Gadol AA, Miller JC (2015). Novel delivery methods bypassing the blood-brain and blood-tumor barriers. Neurosurg Focus.

[8] Pardridge WM (2005). The blood-brain barrier: bottleneck in brain drug development. NeuroRx.

[9] Kemper EM, Boogerd W, Thuis I, Beijnen JH, van Tellingen O (2004). Modulation of the blood–brain barrier in oncology: therapeutic opportunities for the treatment of brain tumours?. Cancer Treat Rev.

[10] Obermeier B, Daneman R, Ransohoff RM (2013). Development, maintenance and disruption of the blood-brain barrier. Nat Med.

[11] Rodriguez A, Tatter S, Debinski W (2015). Neurosurgical techniques for disruption of the blood-brain barrier for glioblastoma treatment. Pharmaceutics.

[12] Erdő F, Denes L, de Lange E (2017). Age-associated physiological and pathological changes at the blood–brain barrier: a review. J Cereb Blood Flow Metab.

[13] Zlokovic BV (2011). Neurovascular pathways to neurodegeneration in Alzheimer’s disease and other disorders. Nat Rev Neurosci.

[14] Sharif Y, Jumah F, Coplan L, Krosser A, Sharif K, Tubbs RS (2018). Blood brain barrier: a review of its anatomy and physiology in health and disease. Clin Anat.

[15] Iadecola C (2017). The neurovascular unit coming of age: a journey through neurovascular coupling in health and disease. Neuron.

[16] Sarmento B (2016). Concepts and models for drug permeability studies. Concepts and models for drug permeability studies: cell and tissue based in vitro culture models.

[17] Masserini M (2013). Nanoparticles for brain drug delivery. ISRN Biochem.

[18] Bednarczyk J, Lukasiuk K (2011). Tight junctions in neurological diseases. Acta Neurobiol Exp.

[19] Hashimoto Y, Campbell M (2020). Tight junction modulation at the blood-brain barrier: current and future perspectives. Biochim Et Biophys Acta BBA Biomembranes.

[20] Wolburg H, Lippoldt A (2002). Tight junctions of the blood–brain barrier. Vascul Pharmacol.

[21] Aird WC (2007). Phenotypic heterogeneity of the endothelium: I. Structure, function, and mechanisms. Circ Res.

[22] Serlin Y, Shelef I, Knyazer B, Friedman A (2015). Anatomy and physiology of the blood-brain barrier. Semin Cell Dev Biol.

[23] Alvarez JI, Cayrol R, Prat A (2011). Disruption of central nervous system barriers in multiple sclerosis. Biochim Et Biophys Acta BBA—Mol Basis Dis.

[24] Frank RN, Keirn RJ, Kennedy A, Frank KW (1983). Galactose-induced retinal capillary basement membrane thickening: prevention by Sorbinil. Invest Ophthalmol Vis Sci.

[25] Agathe F, Yasuhiro N, Yukari SM, Tomomi F, Kaoru S, Matsusaki M (2021). An in vitro self-organized three-dimensional model of the blood-brain barrier microvasculature. Biomed Mater.

[26] Armulik A, Genové G, Mäe M, Nisancioglu MH, Wallgard E, Niaudet C (2010). Pericytes regulate the blood–brain barrier. Nature.

[27] Daneman R, Zhou L, Kebede AA, Barres BA (2010). Pericytes are required for blood-brain barrier integrity during embryogenesis. Nature.

[28] Armulik A, Genové G, Betsholtz C (2011). Pericytes: developmental, physiological, and pathological perspectives, problems, and promises. Dev Cell.

[29] Alvarez JI, Dodelet-Devillers A, Kebir H, Ifergan I, Fabre PJ, Terouz S (2011). The hedgehog pathway promotes blood-brain barrier integrity and CNS immune quiescence. Science.

[30] Abbott NJ, Patabendige AAK, Dolman DEM, Yusof SR, Begley DJ (2010). Neurobiology of disease structure and function of the blood—brain barrier. Neurobiol Dis.

[31] Rodríguez-Arellano JJ, Parpura V, Zorec R, Verkhratsky A (2016). Astrocytes in physiological aging and Alzheimer’s disease. Neuroscience.

[32] Campbell HK, Maiers JL, DeMali KA (2017). Interplay between tight junctions & adherens junctions. Exp Cell Res.

[33] Harris TJC, Tepass U (2010). Adherens junctions: from molecules to morphogenesis. Nat Rev Mol Cell Biol.

[34] Meng W, Takeichi M (2009). Adherens junction: molecular architecture and regulation. Cold Spring Harb Perspect Biol.

[35] Redzic Z (2011). Molecular biology of the blood-brain and the blood-cerebrospinal fluid barriers: similarities and differences. Fluids Barriers CNS.

[36] Williams MJ, Lowrie MB, Bennett JP, Firth JA, Clark P (2005). Cadherin-10 is a novel blood-brain barrier adhesion molecule in human and mouse. Brain Res.

[37] Tietz S, Engelhardt B (2015). Brain barriers: crosstalk between complex tight junctions and adherens junctions. J Cell Biol.

[38] Luissint AC, Artus C, Glacial F, Ganeshamoorthy K, Couraud PO (2012). Tight junctions at the blood brain barrier: physiological architecture and disease-associated dysregulation. Fluids Barriers CNS.

[39] Feldman GJ, Mullin JM, Ryan MP (2005). Occludin: structure, function and regulation. Adv Drug Deliv Rev.

[40] Berndt P, Winkler L, Cording J, Breitkreuz-Korff O, Rex A, Dithmer S (2019). Tight junction proteins at the blood–brain barrier: far more than claudin-5. Cell Mol Life Sci.

[41] Mineta K, Yamamoto Y, Yamazaki Y, Tanaka H, Tada Y, Saito K (2011). Predicted expansion of the claudin multigene family. FEBS Lett.

[42] Nitta T, Hata M, Gotoh S, Seo Y, Sasaki H, Hashimoto N (2003). Size-selective loosening of the blood-brain barrier in claudin-5–deficient mice. J Cell Biol.

[43] Ebnet K, Suzuki A, Ohno S, Vestweber D (2004). Junctional adhesion molecules (JAMs): more molecules with dual functions?. J Cell Sci.

[44] Pardridge WM (2009). Alzheimer’s disease drug development and the problem of the blood-brain barrier. Alzheimers Dement.

[45] Fischer H, Gottschlich R, Seelig A (1998). Blood-brain barrier permeation: molecular parameters governing passive diffusion. J Membr Biol.

[46] Mittapalli RK, Manda VK, Adkins CE, Geldenhuys WJ, Lockman PR (2010). Exploiting nutrient transporters at the blood–brain barrier to improve brain distribution of small molecules. Ther Deliv.

[47] Abbott NJ (2013). Blood–brain barrier structure and function and the challenges for CNS drug delivery. J Inherit Metab Dis.

[48] Gosselet F, Loiola RA, Roig A, Rosell A, Culot M (2021). Central nervous system delivery of molecules across the blood-brain barrier. Neurochem Int.

[49] Saraiva C, Praça C, Ferreira R, Santos T, Ferreira L, Bernardino L (2016). Nanoparticle-mediated brain drug delivery: overcoming blood—brain barrier to treat neurodegenerative diseases. J Control Release.

[50] Sweeney MD, Zhao Z, Montagne A, Nelson AR, Zlokovic Bv (2019). Blood-brain barrier: from physiology to disease and back. Physiol Rev.

[51] Wang D, Wang C, Wang L, Chen Y (2019). A comprehensive review in improving delivery of small-molecule chemotherapeutic agents overcoming the blood-brain/brain tumor barriers for glioblastoma treatment. Drug Deliv.

[52] Miranda A, Blanco-Prieto M, Sousa J, Pais A, Vitorino C (2017). Breaching barriers in glioblastoma. Part I: molecular pathways and novel treatment approaches. Int J Pharm.

[53] Oberoi RK, Parrish KE, Sio TT, Mittapalli RK, Elmquist WF, Sarkaria JN (2016). Strategies to improve delivery of anticancer drugs across the blood–brain barrier to treat glioblastoma. Neuro Oncol.

[54] He Q, Liu J, Liang J, Liu X, Li W, Liu Z (2018). Towards improvements for penetrating the blood-brain barrier—recent progress from a material and pharmaceutical perspective. Cells.

[55] Li J, Zheng M, Shimoni O, Banks WA, Bush AI, Gamble JR (2021). Development of novel therapeutics targeting the blood-brain barrier: from barrier to carrier. Adv Sci.

[56] Kondoh M, Masuyama A, Takahashi A, Asano N, Mizuguchi H, Koizumi N (2005). A novel strategy for the enhancement of drug absorption using a claudin modulator. Mol Pharmacol.

[57] Hashimoto Y, Shirakura K, Okada Y, Takeda H, Endo K, Tamura M (2017). Claudin-5-binders enhance permeation of solutes across the blood-brain barrier in a mammalian model. J Pharmacol Exp Ther.

[58] Hashimoto Y, Okada Y, Shirakura K, Tachibana K, Sawada M, Yagi K (2019). Anti-claudin antibodies as a concept for development of claudin-directed drugs. J Pharmacol Exp Ther.

[59] Nag S, David JB, Kalimo H (2005). Blood brain barrier, exchange of metabolites and gases. BT-pathology and genetics: cerebrovascula.

[60] Barber TW, Brockway JA, Higgins LS (1970). The density of tissues in and about the head. Acta Neurol Scand.

[61] Sharabi S, Last D, Guez D, Daniels D, Hjouj MI, Salomon S (2014). Dynamic effects of point source electroporation on the rat brain tissue. Bioelectrochemistry.

[62] Labuda C, Newman WR, Hoffmeister BK, Chambliss CKM (2022). Two-dimensional mapping of the ultrasonic attenuation and speed of sound in brain. Ultrasonics.

[63] Marceglia S, Mrakic-Sposta S, Rosa M, Ferrucci R, Mameli F, Vergari M (2016). Transcranial direct current stimulation modulates cortical neuronal activity in Alzheimer’s disease. Front Neurosci.

[64] Norton BJ, Bowler MA, Wells JD, Keller MD (2013). Analytical approaches for determining heat distributions and thermal criteria for infrared neural stimulation. J Biomed Opt.

[65] Lim HW, Silpa-archa N, Amadi U, Menter A, Van Voorhees AS, Lebwohl M (2015). Phototherapy in dermatology: a call for action. J Am Acad Dermatol.

[66] Vreman HJ, Wong RJ, Stevenson DK (2004). Phototherapy: current methods and future directions. Semin Perinatol.

[67] Shibu ES, Hamada M, Murase N, Biju V (2013). Nanomaterials formulations for photothermal and photodynamic therapy of cancer. J Photochem Photobiol, C.

[68] Zhang C, Feng W, Vodovozova E, Tretiakova D, Boldyrevd I, Li Y (2018). Photodynamic opening of the blood-brain barrier to high weight molecules and liposomes through an optical clearing skull window. Biomed Opt Express.

[69] Agostinis P, Berg K, Cengel KA, Foster TH, Girotti AW, Gollnick SO (2011). Photodynamic therapy of cancer: an update. CA Cancer J Clin.

[70] Chilakamarthi U, Giribabu L (2017). Photodynamic therapy: past, present and future. Chem Rec.

[71] Gunaydin G, Gedik ME, Ayan S (2021). Photodynamic therapy—current limitations and novel approaches. Front Chem.

[72] Hirschberg H, Peng Q, Uzal FA, Chighvinadze D, Zhang MJ, Madsen SJ. Targeted opening of the blood brain barrier by ALA-mediated photodynamic therapy. In: Kessel DH, editor. 2009. p. 73801C. 10.1002/lsm.20670 PMC2667623 18798293

[73] Hirschberg H, Uzal FA, Chighvinadze D, Zhang MJ, Peng Q, Madsen SJ (2008). Disruption of the blood-brain barrier following ALA-mediated photodynamic therapy. Lasers Surg Med.

[74] Ito S, Rachinger W, Stepp H, Reulen HJ, Stummer W (2004). Oedema formation in experimental photo-irradiation therapy of brain tumours using 5-ALA. Acta Neurochir.

[75] Mii Dii HH, Angell-Petersen E, Spetalen S, Mii Dii MM, Madsen SJ (2007). Increased brain edema following 5-aminolevulinic acid mediated photodynamic in normal and tumor bearing rats.

[76] Zhang C, Zhu D, Tretiakova D, Vodovozova E, Boldyrevd I, Kürths J (2018). Photodynamic opening of the blood-brain barrier to high weight molecules and liposomes through an optical clearing skull window. Biomed Opt Express.

[77] Inglut CT, Gray K, Vig S, Jung JW, Stabile J, Zhang Y (2020). Photodynamic priming modulates endothelial cell-cell junction phenotype for light-activated remote control of drug delivery. IEEE J Sel Top Quantum Electron.

[78] Semyachkina-Glushkovskaya O, Kurths J, Borisova E, Sokolovski S, Mantareva V, Angelov I (2017). Photodynamic opening of blood-brain barrier. Biomed Opt Express.

[79] Ota H, Matsumura M, Miki N, Minamitami H (2009). Photochemically induced increase in endothelial permeablity regulated by RhoA activation. Photochem Photobiol Sci.

[80] Schmidt SI, Blaabjerg M, Freude K, Meyer M (2022). RhoA signaling in neurodegenerative diseases. Cells.

[81] Sporn LA, Foster TH (1992). Photofrin and light induces microtubule depolymerization in cultured human endothelial cells. Cancer Res.

[82] Li B, Zhao WD, Tan ZM, Fang WG, Zhu L, Chen YH (2006). Involvement of Rho/ROCK signalling in small cell lung cancer migration through human brain microvascular endothelial cells. FEBS Lett.

[83] Tojkander S, Gateva G, Lappalainen P (2012). Actin stress fibers—assembly, dynamics and biological roles. J Cell Sci.

[84] Noda K, Zhang J, Fukuhara S, Kunimoto S, Yoshimura M, Mochizuki N (2010). Vascular endothelial-cadherin stabilizes at cell-cell junctions by anchoring to circumferential actin bundles through α- and β-catenins in cyclic AMP-Epac-Rap1 signal-activated endothelial cells. Mol Biol Cell.

[85] Hebda JK, Leclair HM, Azzi S, Roussel C, Scott MGH, Bidère N (2013). The C-terminus region of β-arrestin1 modulates VE-cadherin expression and endothelial cell permeability. Cell Commun Signal.

[86] Ashraf O, Patel NV, Hanft S, Danish SF (2018). Laser-induced thermal therapy in neuro-oncology: a review. World Neurosurg.

[87] Salehi A, Paturu MR, Patel B, Cain MD, Mahlokozera T, Yang AB (2020). Therapeutic enhancement of blood–brain and blood–tumor barriers permeability by laser interstitial thermal therapy. Neurooncol Adv.

[88] Salem U, Kumar VA, Madewell JE, Schomer DF, de Almeida Bastos DC, Zinn PO (2019). Neurosurgical applications of MRI guided laser interstitial thermal therapy (LITT). Cancer Imaging.

[89] Sabel M, Rommel F, Kondakci M, Gorol M, Willers R, Bilzer T (2003). Locoregional opening of the rodent blood-brain barrier for paclitaxel using Nd:YAG laser-induced thermo therapy: a new concept of adjuvant glioma therapy?. Lasers Surg Med.

[90] Leuthardt EC, Duan C, Kim MJ, Campian JL, Kim AH, Miller-Thomas MM (2016). Hyperthermic laser ablation of recurrent glioblastoma leads to temporary disruption of the peritumoral blood brain barrier. PLoS ONE.

[91] Trivedi DP, Hallock KJ, Bergethon PR (2013). Electric fields caused by blood flow modulate vascular endothelial electrophysiology and nitric oxide production. Bioelectromagnetics.

[92] Balança B, Meiller A, Bezin L, Dreier JP, Marinesco S, Lieutaud T (2017). Altered hypermetabolic response to cortical spreading depolarizations after traumatic brain injury in rats. J Cereb Blood Flow Metab.

[93] Lerner EC, Edwards RM, Wilkinson DS, Fecci PE (2022). Laser ablation: heating up the anti-tumor response in the intracranial compartment. Adv Drug Deliv Rev.

[94] Tewari D, Sah AN, Bawari S, Nabavi SF, Dehpour AR, Shirooie S (2020). Role of nitric oxide in neurodegeneration: function, regulation, and inhibition. Curr Neuropharmacol.

[95] Alm P, Sharma HS, Hedlund S, Sjöquist PO, Westman J (1998). Nitric oxide in the pathophysiology of hyperthermic brain injury. Influence of a new anti-oxidant compound H-290/51. A pharmacological study using immunohistochemistry in the rat. Amino Acids.

[96] Yamaguchi T, Shimizu K, Kokubu Y, Nishijima M, Takeda S, Ogura H (2019). Effect of heat stress on blood-brain barrier integrity in iPS cell-derived microvascular endothelial cell models. PLoS ONE.

[97] Gray MT, Woulfe JM (2015). Striatal blood-brain barrier permeability in Parkinson’s disease. J Cereb Blood Flow Metab.

[98] Volkmann J, Albanese A, Antonini A, Chaudhuri KR, Clarke CE, De Bie RMA (2013). Selecting deep brain stimulation or infusion therapies in advanced Parkinson’s disease: an evidence-based review. J Neurol.

[99] Monai H, Ohkura M, Tanaka M, Oe Y, Konno A, Hirai H (2016). Calcium imaging reveals glial involvement in transcranial direct current stimulation-induced plasticity in mouse brain. Nat Commun.

[100] Lok J, Gupta P, Guo S, Kim WJ, Whalen MJ, Van Leyen K (2007). Cell-cell signaling in the neurovascular unit. Neurochem Res.

[101] Shin DW, Fan J, Luu E, Khalid W, Xia Y, Khadka N (2020). In vivo modulation of the blood-brain barrier permeability by transcranial direct current stimulation (tDCS). Ann Biomed Eng.

[102] Wang Y, Hao Y, Zhou J, Fried PJ, Wang X, Zhang J (2015). Direct current stimulation over the human sensorimotor cortex modulates the brain’s hemodynamic response to tactile stimulation. Eur J Neurosci.

[103] Brunoni AR, Nitsche MA, Bolognini N, Bikson M, Wagner T, Merabet L (2012). Clinical research with transcranial direct current stimulation (tDCS): challenges and future directions. Brain Stimul.

[104] Liebetanz D, Koch R, Mayenfels S, König F, Paulus W, Nitsche MA (2009). Safety limits of cathodal transcranial direct current stimulation in rats. Clin Neurophysiol.

[105] Cancel LM, Arias K, Bikson M, Tarbell JM (2018). Direct current stimulation of endothelial monolayers induces a transient and reversible increase in transport due to the electroosmotic effect. Sci Rep.

[106] Xia Y, Li Y, Khalid W, Bikson M, Fu BM (2021). Direct current stimulation disrupts endothelial glycocalyx and tight junctions of the blood-brain barrier in vitro. Front Cell Dev Biol.

[107] Tarbell JM, Demaio L, Mark MZ (1999). Effect of pressure on hydraulic conductivity of endothelial monolayers: role of endothelial cleft shear stress. J Appl Physiol.

[108] Cancel LM, Arias K, Bikson M, Tarbell JM (2018). Direct current stimulation of endothelial monolayers induces a transient and reversible increase in transport due to the electroosmotic effect. Sci Rep.

[109] Bai H, Forrester JV, Zhao M (2011). DC electric stimulation upregulates angiogenic factors in endothelial cells through activation of VEGF receptors. Cytokine.

[110] Lee TH, Avraham H, Lee SH, Avraham S (2002). Vascular endothelial growth factor modulates neutrophil transendothelial migration via up-regulation of interleukin-8 in human brain microvascular endothelial cells. J Biol Chem.

[111] Knotkova H, Nitsche MA, Bikson M, Woods AJ (2019). Practical guide to transcranial direct current stimulation.

[112] Takashima I (2017). Blood-brain barrier derangement after electrical brain stimulation. J Neurol Neuromed.

[113] Salvador E, Kessler AF, Domröse D, Hörmann J, Schaeffer C, Giniunaite A (2022). Tumor treating fields (TTFields) reversibly permeabilize the blood-brain barrier in vitro and in vivo. Biomolecules.

[114] Hjouj M, Last D, Guez D, Daniels D, Sharabi S, Lavee J (2012). MRI study on reversible and irreversible electroporation induced blood brain barrier disruption. PLoS ONE.

[115] Sharabi S, Bresler Y, Ravid O, Shemesh C, Atrakchi D, Schnaider-Beeri M (2019). Transient blood–brain barrier disruption is induced by low pulsed electrical fields in vitro: an analysis of permeability and trans-endothelial electric resistivity. Drug Deliv.

[116] Bonakdar M, Graybill PM, Davalos RV (2017). A microfluidic model of the blood–brain barrier to study permeabilization by pulsed electric fields. RSC Adv.

[117] Lorenzo MF, Thomas SC, Kani Y, Hinckley J, Lee M, Adler J (2019). Temporal characterization of blood-brain barrier disruption with high-frequency electroporation. Cancers.

[118] Partridge BR, Kani Y, Lorenzo MF, Campelo SN, Allen IC, Hinckley J (2022). High-frequency irreversible electroporation (H-FIRE) induced blood-brain barrier disruption is mediated by cytoskeletal remodeling and changes in tight junction protein regulation. Biomedicines.

[119] Partridge B, Eardley A, Morales BE, Campelo SN, Lorenzo MF, Mehta JN (2022). Advancements in drug delivery methods for the treatment of brain disease. Front Vet Sci.

[120] Sun Y, Du L, Yang M, Li Q, Jia X, Li Q (2021). Brain-targeted drug delivery assisted by physical techniques and its potential applications in traditional Chinese medicine. J Tradit Chin Med Sci.

[121] Sharabi S, Kos B, Last D, Guez D, Daniels D, Harnof S (2016). A statistical model describing combined irreversible electroporation and electroporation-induced blood-brain barrier disruption. Radiol Oncol.

[122] Sharabi S, Last D, Daniels D, Fabian ID, Atrakchi D, Bresler Y (2021). Non-invasive low pulsed electrical fields for inducing BBB disruption in mice—feasibility demonstration. Pharmaceutics.

[123] Rajagopalan NR, Vista WR, Fujimori M, Vroomen LGPH, Jiménez JM, Khadka N (2023). Cytoskeletal remodeling and gap junction translocation mediates blood-brain barrier disruption by non-invasive low-voltage pulsed electric fields. Ann Biomed Eng.

[124] Cooper I, Last D, Ravid O, Rand D, Matsree E, Omesi L (2023). BBB opening by low pulsed electric fields, depicted by delayed-contrast MRI, enables efficient delivery of therapeutic doxorubicin doses into mice brains. Fluids Barriers CNS.

[125] Idbaih A, Canney M, Belin L, Desseaux C, Vignot A, Bouchoux G (2019). Safety and feasibility of repeated and transient blood-brain barrier disruption by pulsed ultrasound in patients with recurrent glioblastoma. Clin Cancer Res.

[126] Meng Y, Jones RM, Davidson B, Huang Y, Pople CB, Surendrakumar S (2021). Technical principles and clinical workflow of transcranial MR-guided focused ultrasound. Stereotact Funct Neurosurg.

[127] Beccaria K, Canney M, Goldwirt L, Fernandez C, Piquet J, Perier MC (2016). Ultrasound-induced opening of the blood-brain barrier to enhance temozolomide and irinotecan delivery: an experimental study in rabbits. J Neurosurg.

[128] Davidson B, Hamani C, Huang Y, Jones RM, Meng Y, Giacobbe P (2020). Magnetic resonance-guided focused ultrasound capsulotomy for treatment-resistant psychiatric disorders. Oper Neurosurg.

[129] Sheikov N, McDannold N, Vykhodtseva N, Jolesz F, Hynynen K (2004). Cellular mechanisms of the blood-brain barrier opening induced by ultrasound in presence of microbubbles. Ultrasound Med Biol.

[130] Lipsman N, Meng Y, Bethune AJ, Huang Y, Lam B, Masellis M (2018). Blood–brain barrier opening in Alzheimer’s disease using MR-guided focused ultrasound. Nat Commun.

[131] Coluccia D, Fandino J, Schwyzer L, O’Gorman R, Remonda L, Anon J (2014). First noninvasive thermal ablation of a brain tumor with MR-guided focusedultrasound. J Ther Ultrasound.

[132] Moosa S, Martínez-Fernández R, Elias WJ, del Alamo M, Eisenberg HM, Fishman PS (2019). The role of high-intensity focused ultrasound as a symptomatic treatment for Parkinson’s disease. Mov Disord.

[133] Bunevicius A, McDannold NJ, Golby AJ (2020). Focused ultrasound strategies for brain tumor therapy. Oper Neurosurg.

[134] Mainprize T, Lipsman N, Huang Y, Meng Y, Bethune A, Ironside S (2019). Blood-brain barrier opening in primary brain tumors with non-invasive MR-guided focused ultrasound: a clinical safety and feasibility study. Sci Rep.

[135] Abrahao A, Meng Y, Llinas M, Huang Y, Hamani C, Mainprize T (2019). First-in-human trial of blood–brain barrier opening in amyotrophic lateral sclerosis using MR-guided focused ultrasound. Nat Commun.

[136] Fini M, Tyler WJ (2017). Transcranial focused ultrasound: a new tool for non-invasive neuromodulation. Int Rev Psychiatry.

[137] Jalali S, Huang Y, Dumont DJ, Hynynen K (2010). Focused ultrasound-mediated bbb disruption is associated with an increase in activation of AKT: Experimental study in rats. BMC Neurol.

[138] Ting CY, Fan CH, Liu HL, Huang CY, Hsieh HY, Yen TC (2012). Concurrent blood-brain barrier opening and local drug delivery using drug-carrying microbubbles and focused ultrasound for brain glioma treatment. Biomaterials.

[139] Wei KC, Chu PC, Wang HYJ, Huang CY, Chen PY, Tsai HC (2013). Focused ultrasound-induced blood-brain barrier opening to enhance temozolomide delivery for glioblastoma treatment: a preclinical study. PLoS ONE.

[140] Liu HL, Huang CY, Chen JY, Wang HYJ, Chen PY, Wei KC (2014). Pharmacodynamic and therapeutic investigation of focused ultrasound-induced blood-brain barrier opening for enhanced temozolomide delivery in glioma treatment. PLoS ONE.

[141] Horodyckid C, Canney M, Vignot A, Boisgard R, Drier A, Huberfeld G (2017). Safe long-term repeated disruption of the blood-brain barrier using an implantable ultrasound device: a multiparametric study in a primate model. J Neurosurg.

[142] Chen H, Konofagou EE (2014). The size of blood-brain barrier opening induced by focused ultrasound is dictated by the acoustic pressure. J Cereb Blood Flow Metab.

[143] Alonso A, Reinz E, Jenne JW, Fatar M, Schmidt-Glenewinkel H, Hennerici MG (2010). Reorganization of gap junctions after focused ultrasound blood-brain barrier opening in the rat brain. J Cereb Blood Flow Metab.

[144] Jordão JF, Thévenot E, Markham-coultes K, Scarcelli T, Weng Qi Y, Xhima K (2013). Amyloid- β plaque reduction, endogenous antibody delivery and glial activation by brain-targeted, transcranial focused ultrasound. Exp Neurol.

[145] Lin CY, Hsieh HY, Pitt WG, Huang CY, Tseng IC, Yeh CK (2015). Focused ultrasound-induced blood-brain barrier opening for non-viral, non-invasive, and targeted gene delivery. J Control Release.

[146] Sheikov N, McDannold N, Sharma S, Hynynen K (2008). Effect of focused ultrasound applied with an ultrasound contrast agent on the tight junctional integrity of the brain microvascular endothelium. Ultrasound Med Biol.

[147] Choi JJ, Feshitan JA, Baseri B, Wang S, Tung YS, Borden MA (2010). Microbubble-size dependence of focused ultrasound-induced blood-brain barrier opening in mice in vivo. IEEE Trans Biomed Eng.

[148] Vlachos F, Tung YS, Konofagou E (2011). Permeability dependence study of the focused ultrasound-induced blood-brain barrier opening at distinct pressures and microbubble diameters using DCE-MRI. Magn Resonance Med.

[149] Samiotaki G, Vlachos F, Tung YS, Konofagou EE (2012). A quantitative pressure and microbubble-size dependence study of focused ultrasound-induced blood-brain barrier opening reversibility in vivo using MRI. Magn Resonance Med.

[150] Aryal M, Arvanitis CD, Alexander PM, McDannold N (2014). Ultrasound-mediated blood-brain barrier disruption for targeted drug delivery in the central nervous system. Adv Drug Deliv Rev.

[151] VanBavel E (2007). Effects of shear stress on endothelial cells: possible relevance for ultrasound applications. Prog Biophys Mol Biol.

[152] Krizanac-Bengez L, Mayberg MR, Janigro D (2004). The cerebral vasculature as a therapeutic target for neurological disorders and the role of shear stress in vascular homeostatis and pathophysiology. Neurol Res.

[153] Hoger JH, Ilyin VI, Forsyth S, Hoger A (2002). Shear stress regulates the endothelial Kir21 ion channel. Proc Natl Acad Sci.

[154] Walsh TG, Murphy RP, Fitzpatrick P, Rochfort KD, Guinan AF, Murphy A (2011). Stabilization of brain microvascular endothelial barrier function by shear stress involves VE-cadherin signaling leading to modulation of pTyr-occludin levels. J Cell Physiol.

[155] Taddei A, Giampietro C, Conti A, Orsenigo F, Breviario F, Pirazzoli V (2008). Endothelial adherens junctions control tight junctions by VE-cadherin-mediated upregulation of claudin-5. Nat Cell Biol.

[156] Tzima E, Irani-Tehrani M, Kiosses WB, Dejana E, Schultz DA, Engelhardt B (2005). A mechanosensory complex that mediates the endothelial cell response to fluid shear stress. Nature.

[157] Seteikin AYu, Krasnikov IV, Drakaki E, Makropoulou M (2013). Dynamic model of thermal reaction of biological tissues to laser-induced fluorescence and photodynamic therapy. J Biomed Opt.

[158] Garcia PA, Rossmeisl JH, Robertson JL, Olson JD, Johnson AJ, Ellis TL (2012). 7.0-T magnetic resonance imaging characterization of acute blood-brain-barrier disruption achieved with intracranial irreversible electroporation. PLoS ONE.

[159] Barzegar-Fallah A, Gandhi K, Rizwan SB, Slatter TL, Reynolds JNJ (2022). Harnessing ultrasound for targeting drug delivery to the brain and breaching the blood-brain tumour barrier. Pharmaceutics.

[160] https://clinicaltrials.gov/.

[161] Wasielewska JM, White AR (2022). Focused ultrasound-mediated drug delivery in humans—a path towards translation in neurodegenerative diseases. Pharm Res.

